# Machine Learning Approaches for Hospital Acquired Pressure Injuries: A Retrospective Study of Electronic Medical Records

**DOI:** 10.3389/fmedt.2022.926667

**Published:** 2022-06-16

**Authors:** Joshua J. Levy, Jorge F. Lima, Megan W. Miller, Gary L. Freed, A. James O'Malley, Rebecca T. Emeny

**Affiliations:** ^1^Department of Epidemiology, Geisel School of Medicine at Dartmouth, Hanover, NH, United States; ^2^Department of Pathology, Dartmouth Hitchcock Medical Center, Lebanon, NH, United States; ^3^Quantitative Biomedical Sciences, Geisel School of Medicine at Dartmouth, Hanover, NH, United States; ^4^Department of Wound Care Services, Dartmouth Hitchcock Medical Center, Lebanon, NH, United States; ^5^Department of Plastic Surgery, Dartmouth Hitchcock Medical Center, Lebanon, NH, United States; ^6^Department of Biomedical Data Science, Geisel School of Medicine at Dartmouth, Hanover, NH, United States; ^7^The Dartmouth Institute for Health Policy and Clinical Practice, Geisel School of Medicine at Dartmouth, Hanover, NH, United States

**Keywords:** machine learning, artificial intelligence, electronic medical records, hospital acquired pressure injuries, interpretability

## Abstract

**Background:**

Many machine learning heuristics integrate well with Electronic Medical Record (EMR) systems yet often fail to surpass traditional statistical models for biomedical applications.

**Objective:**

We sought to compare predictive performances of 12 machine learning and traditional statistical techniques to predict the occurrence of Hospital Acquired Pressure Injuries (HAPI).

**Methods:**

EMR information was collected from 57,227 hospitalizations acquired from Dartmouth Hitchcock Medical Center (April 2011 to December 2016). Twelve classification algorithms, chosen based upon classic regression and recent machine learning techniques, were trained to predict HAPI incidence and performance was assessed using the Area Under the Receiver Operating Characteristic Curve (AUC).

**Results:**

Logistic regression achieved a performance (AUC = 0.91 ± 0.034) comparable to the other machine learning approaches. We report discordance between machine learning derived predictors compared to the traditional statistical model. We visually assessed important patient-specific factors through Shapley Additive Explanations.

**Conclusions:**

Machine learning models will continue to inform clinical decision-making processes but should be compared to traditional modeling approaches to ensure proper utilization. Disagreements between important predictors found by traditional and machine learning modeling approaches can potentially confuse clinicians and need to be reconciled. These developments represent important steps forward in developing real-time predictive models that can be integrated into EMR systems to reduce unnecessary harm.

## Introduction

Hospital Acquired Pressure Injuries (HAPI) are preventable medical errors with costly implications for patients, health care institutions and consumers (1). These injuries arise from a sustained period of compression between a bony surface and an external surface, often due to immobility and shear (2). The development and occurrence of these events are difficult to detect and localize during early stages due to little superficial presentation and thus provide further motivation for the development of methods that are able to detect and preempt occurrence of HAPIs (3).

Reported rates of HAPIs vary considerably across the United States, which is largely attributed to inappropriate coding and underreporting. Despite the inability to precisely pinpoint the burden of this condition, a prior study from 2012 has indicated that HAPIs have cost the US healthcare system an estimated 6–15 billion dollars per year (4). Most of these costs have been shifted to hospitals, but patients bear additional liability when factoring for deductibles, co-payments and coinsurance and the additional length of stay needed to treat this condition (5).

Thus, these individual and societal burdens may be reduced by better understanding patient-specific factors associated with HAPI and by using information regularly collected in electronic medical records to develop predictive risk models for prevention of HAPIs. The ability of prediction models to fit a set of data can be evaluated and compared by taking note of the concordance index, otherwise known as the C-statistic or alternatively the area under the receiver operating characteristic curve (AUROC/AUC). The receiver operating characteristic curve explores changes in the model's sensitivity and specificity as the predictive threshold for assignment to the positive class (or outcome, i.e., a HAPI event) is changed (6). In this application, the AUC of the fitted model estimates the probability that a randomly selected hospital encounter that resulted in a HAPI event has a greater predictive probability than a randomly selected hospital encounter without a HAPI event. The larger the C-statistic, the better a model is at discriminating an adverse event (e.g., a HAPI event) from the lack thereof (e.g., non HAPI events).

A well-known clinical predictor of HAPIs is the Braden Scale, a measure that incorporates information from six sub-scales (sensory perception, moisture, activity, mobility, nutrition, and friction/shear) to arrive at a risk score between 6 and 23, where scores below 9 indicate severe risk (7). Prior studies that utilized this scoring system yielded C-statistics of 0.67 and 0.77 (8, 9). Nevertheless, the reported low specificity of the measure begs the inclusion of other important predictors. This has led to the expansion and critical evaluation of the covariates sought to predict HAPI incidence (8).

Machine learning, the specification of a model after a heuristic search for the ideal set of non-linear interactions between predictors, may be a useful tool that can enhance clinical encounters for the prediction and reduction of patient risk (10). Recently, some of these HAPI predictors have been incorporated into logistic regression and machine learning approaches. A recent study applied six diverse machine learning algorithms to a cohort of 7,717 Intensive Care Unit (ICU) patients and reported a C-statistic of 0.83 (11), while another study reported a C-statistic of 0.84 for a general hospital population of 8,286 observations using logistic regression with under-sampling of the control patients during model fitting (12). Other studies include: (1) application of Bayesian Network approaches to the aforementioned cohort of 7,717 ICU patients, achieving a similar C-statistic of 0.83 as before, while improving sensitivity and adding model interpretation through modeling of related risk factors (e.g., medications, diagnoses, and Braden scale factors) (13), (2) a random forest model which leveraged predictors curated from clinical input and previous literature to predict stage 1 HAPI and above with a C-statistic of 0.79 (14), (3) another logistic regression which leveraged ICU-specific features to obtain a recall of 0.74 (15), and (4) other modeling approaches built off of Electronic Medical Records (EMR) and claims data, an online AI platform and another logistic regression model (after comparison between six machine learning methods), obtaining a C-statistic of 0.84 and recall of 0.67, respectively (16, 17). In the Supplementary Material, we have included a table which summarizes these studies for the purpose of comparison to the current study's findings (Supplementary Table 1).

Many of these studies attempt to utilize sophisticated machine learning models without critically evaluating whether it is a more appropriate model than traditional statistical techniques that are more readily adoptable by clinicians. Some studies do not include a traditional statistical model baseline (14), while others appear to neglect the implications of the failure to outperform these traditional techniques (11). In some cases, inappropriate predictors (e.g., those that occur or that are measured in the future) have been included in machine learning models by implementers focused on predictive accuracy to such a degree that they bypass questioning whether their model makes clinical sense. In addition to this, none of these models offer/provide intuitive explanations for predicted risk scores for individual patients (i.e., important risk factors for a given patient but not necessarily important across the entire population), instead reporting global associations; individual-level information could better inform the clinician's treatment of a specific patient, thereby reducing these costly medical errors.

We wanted to apply machine learning techniques to one example of patient outcomes, pressure injury prevention, to demonstrate the utilities of such approaches and illustrate the importance of individual-level model explanations. Here, we improve on previous analytical benchmarks through the rigorous evaluation of a diverse set of machine learning and traditional statistical methods. We arrive at a prediction model that can be understood clearly at the individual level and explains the heterogeneity in the patient population to serve as grounds for the development of future personalized real-time predictive models. Finally, based on our results, we critically assess the role of machine learning for the development of retrospective HAPI prediction models. Nonetheless, these methods may augment standard modeling approaches when evaluating real-time prospective data captured through the EMR.

## Methods

### Methods Overview

**Figure 1:**
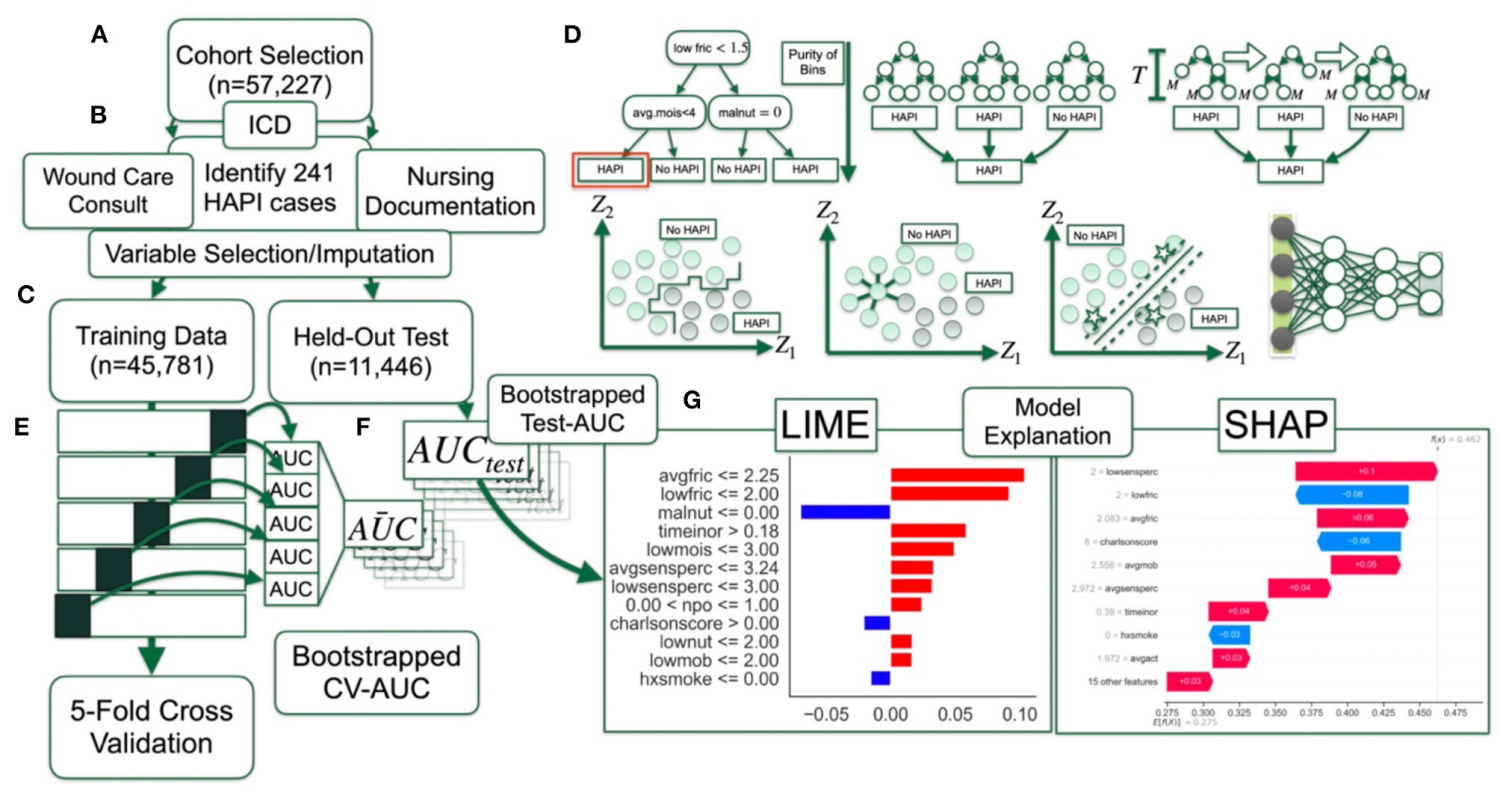
Method overview flow diagram: **(A)** Selection of patient cohort; **(B)** identification of HAPI cases through ICD codes, wound care consults and nursing documentation; data preprocessing with help from; **(C)** training dataset, which comprises 80% of the cohort, whereas 20% of the patients are reserved for final testing; **(D)** 12 machine learning models are trained and finetuned (i.e., hyperparameter scan) using; **(E)** 5-fold cross validation on partitioned training dataset, with average AUC statistics across the folds bootstrapped by patient; **(F)** AUC statistics calculated on held-out test set; **(G)** model interpretation with LIME and SHAP.

A brief overview of the dataset and methods used to train, validate, test and interpret the compared machine learning models for the task of HAPI prediction can be found in Table 1. A visual description (flow chart) of these methods can be found in Figure 1.

**Table 1:** Overview of methods for HAPI prediction and interpretation.

**Task**	**Description**
Data collection	57,227 hospitalizations, 241 positive HAPI cases identified from ICD codes, nursing documentation, wound care consults
Data preprocessing	• Selection of EMR variables based on prior literature, expert opinion, criteria from previous study • Imputation with MICE
Dataset partitioning	• 80% training (*n* = 45,781), further subdivided into five cross validation folds • 20% held-out testing (*n* = 11,446)
Modeling approaches	12 machine learning models (including logistic regression)
Model training and validation	• 5-fold cross validation grid search for selection of hyperparameters • Non-parametric bootstrapping for average cross validation AUC comparison between selected models
Model testing	Non-parametric bootstrapping of test set AUC for final model comparison; ROC visualization
Model interpretation on individual level	• Application of SHAP to identify patient specific salient predictors • Comparison to LIME on few select cases
Global model interpretation	• Aggregate SHAP values across test set patients • Non-parametric bootstrapping of individual Shapley values to compare global importances between models; comparison utilizes spearman correlation and rank biased overlap

### Data Collection, Variable Selection, and Preprocessing

The data utilized for our predictive models were acquired from a prior retrospective study conducted at Dartmouth Hitchcock Medical Center from April 2011 to December 2016 (8) after approval from their Institutional Review Board. Data was collected from EMR for patients who were 18 years or older; each observation represented an individual's hospital stay of 3 or more days and at least 3 recorded Braden scale measurements.

Previous works on HAPI prediction models have only utilized International Classification of Diseases codes (ICD)—the primary means to document medical information such as disease diagnoses, comorbidities, injuries, and inpatient procedures during patient encounters (ICD-9 and ICD-10 represent the 9th and 10th medical classification systems with mappable terminology)—to identify pressure injuries. However, HAPIs are primarily recorded in nursing records, which may not reflect billing codes, and prior research has demonstrated moderately high false positives (pressure injury ICD code but no nursing documentation) and false negatives (nursing documentation but no pressure injury ICD code) (18). Additionally, injuries are often not correctly classified as present on admission (ICD codes do not designate whether the injury was hospital acquired) and this can lead to an inflated number of injuries due to lack of discrimination in coding (18). In light of suboptimal reliability of utilizing ICD coding alone, and our own prior experience, we reduced the number of false positive cases through the following stringent search criteria: presence of ICD-9 (707 range for chronic ulcer of the skin caused by pressure) or ICD-10 (L89 range which is defined as pressure injury) codes, supporting nursing documentation (e.g., injury report in nursing charts) and whether the patient had received consultation by a wound care team (1, 8). Additionally, we removed cases that were present on admission. An identified HAPI case must be at least a stage one and demonstrate all of the aforementioned search criteria. A wound care nurse assisted in the diagnosis via an event reporting system. HAPI identification was in accordance with the National Pressure Injury Advisory Panel Criteria. This constituted a dataset of 57,227 hospitalizations, containing only 241 positive HAPI cases, which epitomizes the highly imbalanced datasets commonly encountered in the diagnosis of rare infections.

We have also included a percentage breakdown of HAPI stage from events reported between 2015 and 2018 and whether the HAPI was medical device related (Supplementary Tables 2, 3), though the latter dataset was collected for a year after our study's data collection period, from 2017 to 2018, and may not be representative of our subpopulation. These breakdowns were based on available data; a complete breakdown of patient-specific characteristics that overlap with our cohort can be found in a recent work (1, 8).

EMR variables were selected for our study based on prior literature, expert opinion and based off of selection criteria from a previous study (8) (Supplementary Tables 4, 5). All individual predictors demonstrated statistically significant associations with HAPIs (Supplementary Table 5), save for ambulatory status and race. We recapitulated the previously reported modeling results (8) to validate our variable selection; however, we removed the length of stay (LOS) variable because it is not valid for use in a task of predicting an outcome from an interim point of a patient's stay as it is not known until the patient is discharged. We imputed two variables with missing data (Supplementary Figure 1); time in operating room (OR) was imputed with zeros under the assumption that a non-record was never present in the OR, and body mass index was imputed using Multiple Imputation by Chained Equations (MICE) (19). The data was split into 80% training (*n* = 45,781) to update the model parameters and 20% testing (*n* = 11,446) for analysis of the ability of the model to generalize to an unseen population. A detailed explanation of the selected variables is included in Supplementary Table 4.

### Description of Modeling Approaches

We performed rigorous evaluations of 12 different predictive modeling approaches: five popular approaches commonly used for HAPI prediction—(1) Naïve Bayes, (2) Decision Trees, (3) Random Forest, (4) XGBoost, and (5) Logistic Regression, and seven additional approaches to complement these assessments as a representative set of machine learning approaches—(6) Linear Discriminant Analysis, (7) Quadratic Discriminant Analysis, (8) Neural Networks (Multilayer Perceptron), (9) Support Vector Machine, (10) K-Nearest Neighbors, and two Bayesian methods to attempt to account for rare events: (11) Bayesian Logistic Regression, and (12) Bayesian Additive Regression Trees (BART) (Supplementary Table 6) (20–29). We estimated the ideal set of model tuning heuristics for all machine learning modeling approaches using an exhaustive grid search on the training set with 5-fold cross-validation. Then, we trained the final predictive model on the training set (including training/validation folds) for all 12 approaches for evaluation on a held-out test set. The primary metric to assess model performance across a wide range of sensitivity thresholds was the area under the receiver operating characteristic (AUC) curve. We have included a discussion of each of these analytical techniques in Sections “Further Description of Analytical Approaches,” “Additional Comparison Approaches” for brief descriptions on all 12 modeling approaches, and Supplementary Table 6 of Supplementary Material (20–24, 30, 31). Statistical comparisons (i.e., AUC differences) between approaches were made across the five cross validation folds (averaged cross validation AUC; CV-AUC) after selection of optimal tuning hyperparameters, and separately on the held-out test set (test AUC). Significance was assessed using non-parametric bootstrapping. Patients within cross validation folds and the held-out test set were resampled with replacement 1,000 times, making sure for each bootstrap iteration that the same set of patients were assessed by all modeling approaches to enable the calculation of AUC differences per iteration. From the non-parametric bootstrapping, we calculated 95% confidence intervals for all comparisons. The difference between algorithmic performance was significant if 0 (no AUC difference) lied outside this interval.

As aforementioned, there are only 241 HAPI-positive samples in a dataset of 57,227 samples. We implemented class balancing techniques to account for rare events, as detailed in Section “Circumventing Class Imbalance Issues” of Supplementary Material (12, 32, 33). When partitioning the dataset into the cross-validation folds and test datasets, we ensured the proportion of HAPI cases to controls were preserved in each fold/dataset. We provide additional information on steps taken to limit bias and the potential for overfitting (i.e., memorizing the training data; does not generalize to unseen data) in Section “Details on Approaches to Limit Overfitting and Hyperparameter Scans” and Supplementary Table 7 of Supplementary Material, including a detailed overview of selected hyperparameters.

### Developing Individual Level Explanations

Concerns about the transparency of machine learning techniques have been raised by researchers and professionals working in highly regulated environments such as in the practice of law and medicine (34). While high predictive accuracy is important, understanding how an algorithm makes a recommendation is fundamental to establish trust and foster acceptance. Many “black box” machine learning models have difficulties in isolating associations between the predictors and outcome. The ability to explain predictions in real world applications is paramount to the actual use and applications for HAPI predictions. While a number of explainability techniques seek to find important predictors across all patients as a way to demonstrate how the model is learning, very few methodologies have been developed to explain which variables had values that placed a given patient at high risk. Additionally, some predictor importance techniques, such as the mean decrease in impurity utilized by random forest methodologies (Section “Further Description of Analytical Approaches” in Supplementary Material), present biased interpretations.

Here, we utilized Shapley Additive Explanations (SHAP) (35) to directly indicate the contribution of each predictor to the predicted probability of being associated with a HAPI event. SHAP estimates a linear model for each held-out observation under scrutiny, where the importance of each predictor is given by the unique model coefficients. However, these personalized models, when summing their coefficients across the cohort, are able to find the overall importance of each predictor. We compared global importances between Random Forest, XGBoost and Logistic Regression with spearman's correlation coefficient (overall association in importances) and rank biased overlap (RBO; agreement measure which places greater importance on top predictors), with 1,000 sample non-parametric bootstrapping of SHAP values (same set of patients for each bootstrap iteration) prior to the calculation of global importances to estimate 95% confidence intervals and communicate the significance of the findings (36). While the SHAP importance from a linear modeling approach should exhibit properties of the linear model, SHAP scores for machine learning models indicate variables that are important and specific to each patient. Plots that summarize the behavior of the model predictors over the entire dataset could offer an insightful tool for aiding the clinician to quickly interpret patient symptoms and intervene to prevent HAPI from occurring. We did not include a detailed analysis of other popular model interpretation approaches (e.g., Local Interpretable Model Explanations, LIME, counterfactual explanations like Anchor and Diverse Counterfactual Explanations, and DiCE) (37–40). We did include a visual comparison between display outputs of SHAP and LIME for a few randomly selected cases. The reason for not opting for these other approaches was in part because SHAP offers both local (i.e., patient-specific) and global (i.e., across patients) interpretations of predictor importances, has demonstrated mathematical guarantees over methods like LIME, and is currently one of the most accepted interpretation techniques (though not without limitations, see Section “Discussion”).

### Code Availability

The results were derived using a custom data pipeline that utilized Jupyter Notebook version 5.7.8 with a Python 3.7.3 Kernel which utilized the *scikit-learn, xgboost, shap, lime*, and *pymc* (for Bayesian approaches) Python libraries (41). The model graphics were generated using the SHAP library. We tested for possible interaction effects using the *InteractionTransformer* package (42). While we are unable to release the data utilized in this study due to patient privacy concerns, we have provided the code used to compare these modeling approaches in the following GitHub repository: https://github.com/jlevy44/DH_Pressure_Injury_Prediction. We have also included supplementary code which enumerates additional comparisons in this repository through the inclusion of two Jupyter notebooks.

## Results

### Classifier Performance

**Figure 2:**
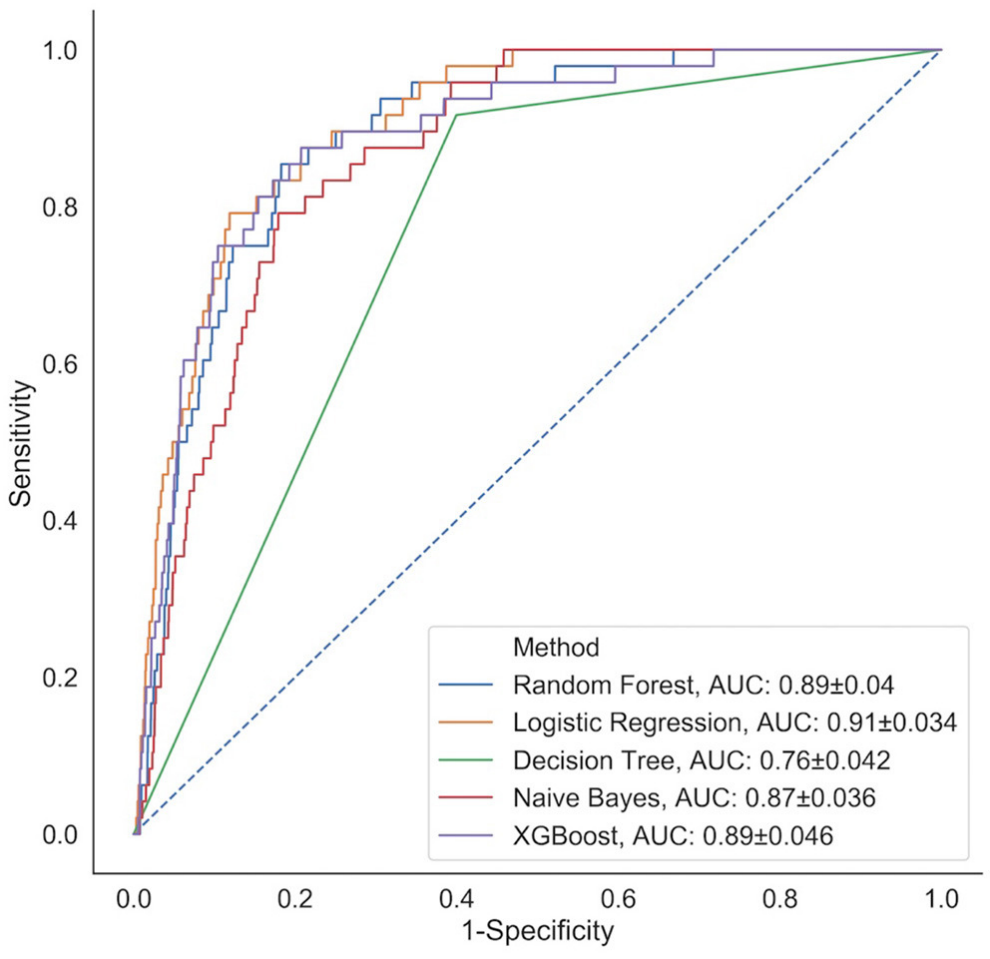
Comparison of classification performance of five analytical models (representative subset of 12 approaches selected) *via* ROC curves calculated on the held-out test set; a similar plot for the remaining prediction models can be found in Supplementary Figure 2.

We fit the 12 modeling approaches to our HAPI dataset and derived C-statistics on the cross-validation folds and held-out test set (Figure 2). The cross-validation C-statistics did not change significantly between validation folds, indicating unbiased partitioning of data for this study (Supplementary Tables 8, 9). Out of all of the models, k-nearest neighbors and decision trees performed the worst with C-statistics of 0.75 and 0.76, respectively, followed by Naïve Bayes with an AUC of 0.87. Results indicate that the logistic regression model (AUC = 0.91) performs comparably to most of the other modeling approaches (e.g., performance comparable to Random Forest, XGBoost AUC = 0.89; the remaining performance statistics for other modeling approaches can be found in Supplementary Tables 10, 11; Supplementary Figure 2). These results provide supporting evidence that the logistic regression model identifies the model specification closest to the underlying true model as other machine learning models failed to surpass the performance of this model, in this particular clinical setting.

### SHAP Comparisons

We applied the SHAP methodology to find the overall important global variables that were important for the prediction of the logistic regression, XGBoost and Random Forest models. While we found a significantly strong positive correlation between the importance of the predictors across all three models (Supplementary Tables 12–14), we noted important disagreements between predictors identified by each model with regards to their level of importance. For instance, low nutrition, average activity and moisture were found to be highly important by the Logistic Regression model, but not by the Random Forest or XGBoost models. Alternatively, smoking was upweighted by the Random Forest and XGBoost models, but not by Logistic Regression. All models found low friction, average mobility and whether the patient's diet was taken by mouth (NPO status) to be important.

**Figure 3:**
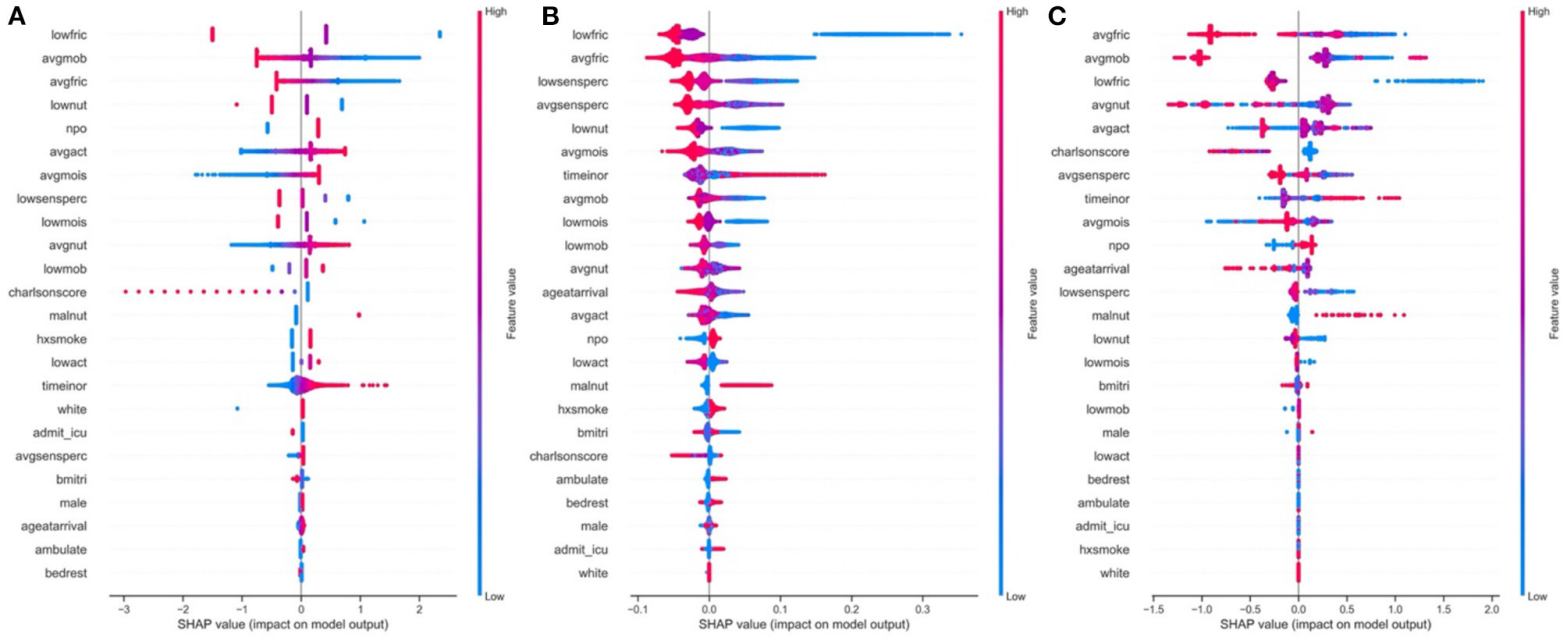
Global predictor importance (SHAP summary plots) of patient specific factors for: **(A)** Logistic Regression, **(B)** Random Forest, **(C)** XGBoost; The plots for each model **(A–C)** consist of a point per patient hospitalization across all predictors. The points are colored by the features value and lateral displacement from the centerline indicates the importance of that feature for that particular individual. Values that increase the probability of being classified as a HAPI are displayed to the right of the centerline of each plot; red dots indicate a high feature value, while blue dots indicate a low feature value. For instance, increased HAPI incidence was associated with decreases in the Braden subscale score for low friction, average mobility, average friction and low nutrition in the logistic regression plot **(A)**.

**Figure 4:**
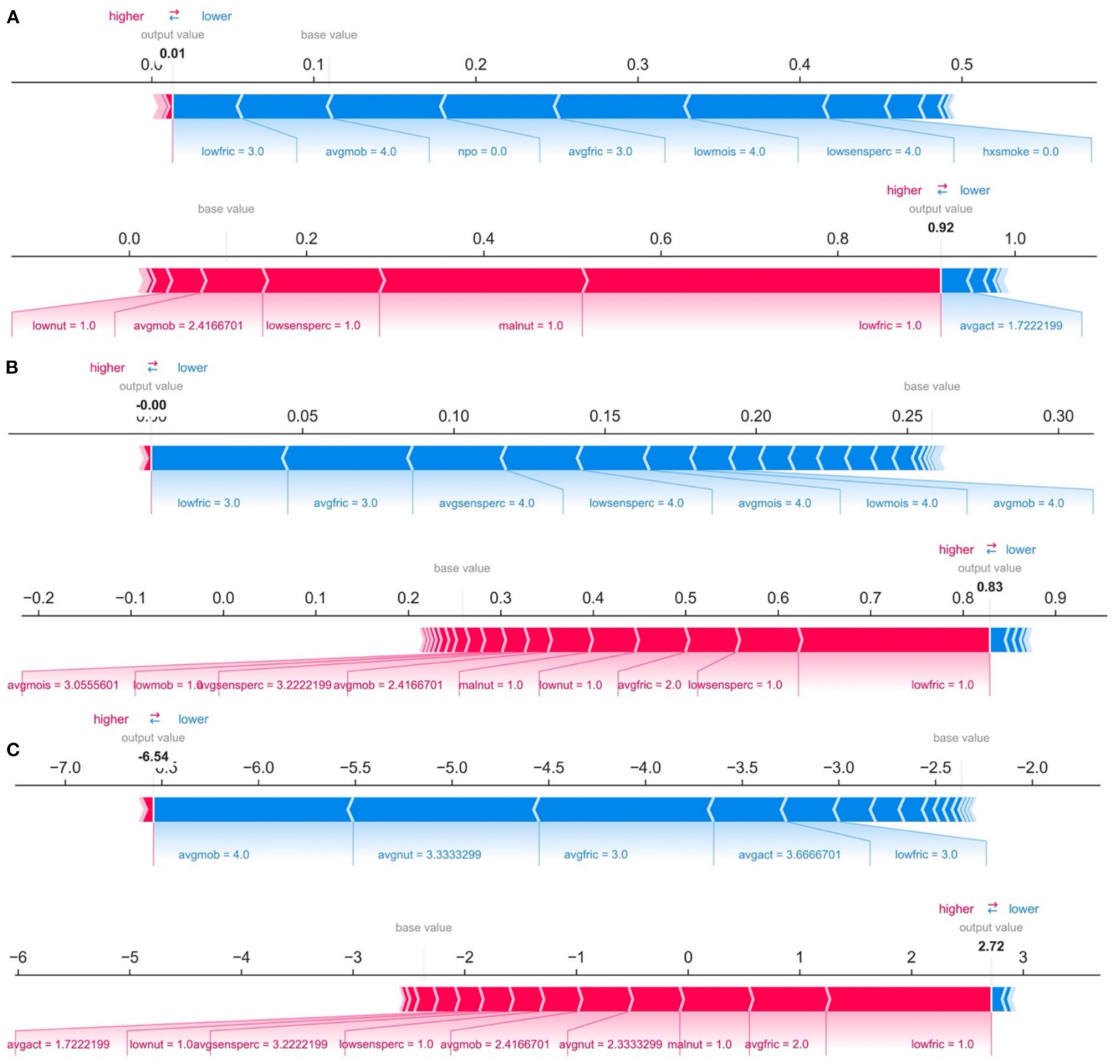
Predictions and decomposition of predictor importance (force plots) for two individuals (top vs. bottom of each panel) using: **(A)** Logistic Regression, **(B)** Random Forest, and **(C)** XGBoost. The predictors are associated with both increased and decreased HAPI. Certain values (e.g., increasing values) may be associated with one or the other. Blue colors indicate predictors that are associated with decreased HAPI incidence, while red colors indicate predictors associated with increased HAPI incidence; magnitude of each arrow indicates the level of importance of the predictor for that prediction.

**Figure 5:**
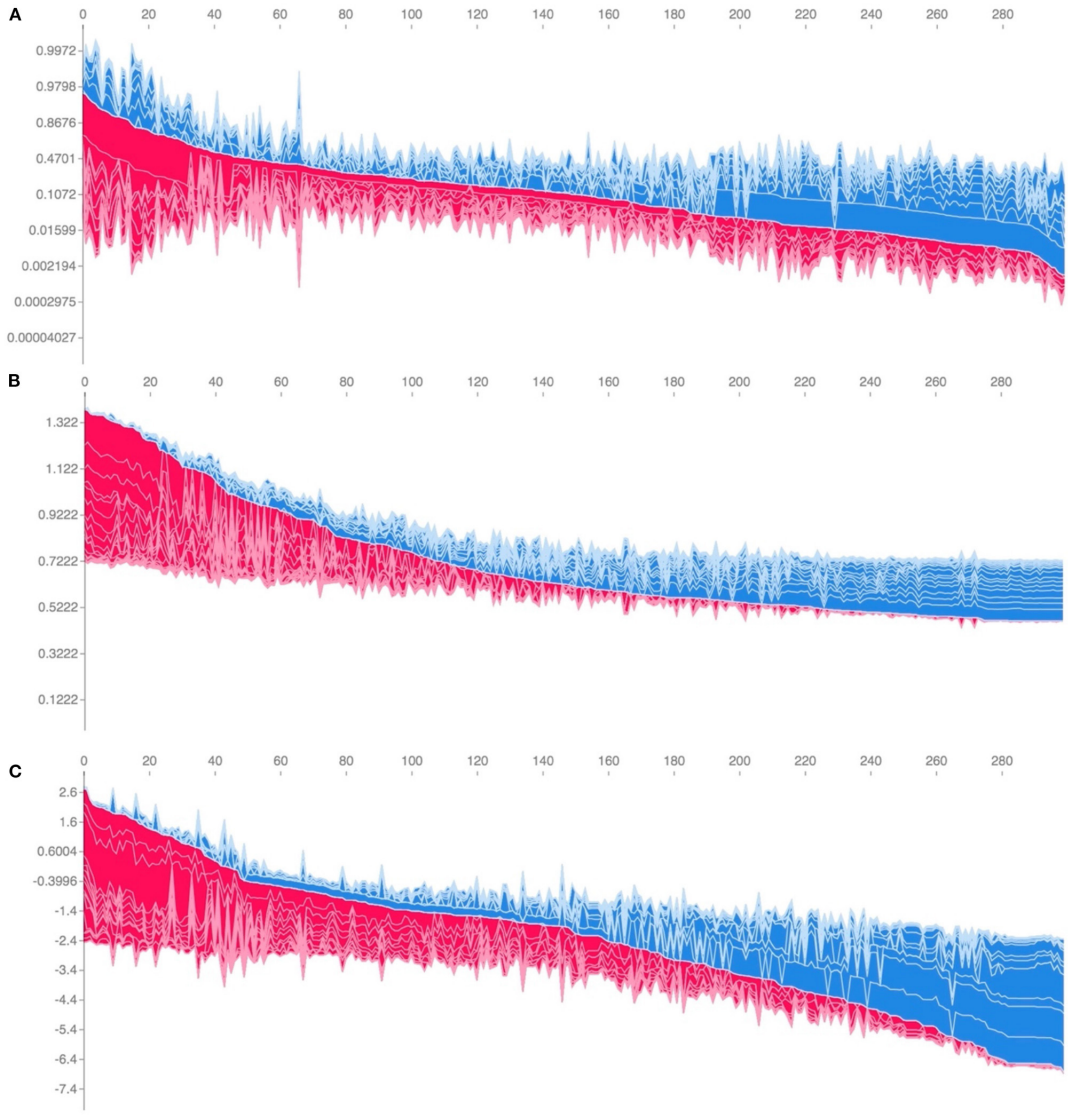
Individual-patient explanations (force-plots) are rotated to a vertical position and stacked horizontally to form interactive plots detailing explanations of HAPI predictions across a large patient population while still allowing interrogation of each patient. We note here that this feature is a web-based interactive plot; the physician or end-user can hover over individuals with their computer mouse, from which the application will display/highlight the important predictors for those individuals. SHAP derived force plots depicting individual predictions and explanations for the first 300 hospitalizations in the study population, ordered from highest HAPI predicted probability (red) to lowest (blue) for: **(A)** Logistic Regression, **(B)** Random Forest, and **(C)** XGBoost (on log-odds scale).

While ranking of important predictors can be found in the SHAP summary plots (Figure 3), one useful feature of SHAP, irrespective of modeling approach, is to portray the important predictors that influence the prediction of a given patient. To more closely interrogate the predictive model for individual patients, we assessed a few select force plots (Figure 4) that depict each model's prediction and the predictors' importance across select individuals. The logistic regression, random forest and XGBoost models all appear to make similar predictions and find similar features to be important for the two observations chosen for display. We have included a figure that showcases the use of this to capture important predictors across 300 patients out of the entire study population (Figure 5). This figure is a static representation of a web-based application that the physician or end-user can interact with to reveal the important predictors for each patient. We additionally compared the display outputs to that of other interpretation techniques that are patient specific (LIME) (Supplementary Figure 3). While for some randomly selected cases, selected predictors appeared to agree visually in both magnitude and direction (Supplementary Figures 3A–D), we noted instances where predictor importances differed (Supplementary Figures 3E,F).

Averaging the absolute value of the SHAP scores for each predictor across the cohort derives an overall importance ranking of the predictors. We found that averaging the SHAP importance values for the logistic regression model yields an approximation of the standardized regression coefficients (Pearson-r = 0.914, *P* < 0.001, average absolute difference = 0.08) (Supplementary Tables 15, 16; Supplementary Figure 4). This convergence reinforces the notion of correspondence between the totaled SHAP coefficients across all of the individuals and the effect estimates of the Logistic Regression model, where the logistic regression effect estimates serve as gold standard values in the case when the logistic regression model represents the true associations.

## Discussion

Machine learning will likely continue to be incorporated into the clinic and inform clinical decision making. Its popularity can be attributed to the promises of better handling large, unstructured, and heterogeneous datasets. We sought to understand how to best utilize these machine learning approaches through extension of its application to pressure injury prevention. As such, our study sought to compare the predictive performance of machine learning and traditional statistical modeling techniques for HAPIs. We built a predictive risk model for hospital acquired pressure injuries based on a retrospective cohort of over 57,000 hospitalizations over a 5-year study period. Our results indicate that performance of the Logistic Regression technique was comparable to the 11 other machine learning approaches when applied to retrospective data without temporal changes in patient status. This ideal model specification (0.91 C-statistic) exceeded the performance recorded in prior publications (0.84 C-statistic) and presents opportunities for early detection of symptoms while minimizing the burden on the clinical staff.

The fact that Logistic Regression was able to achieve such remarkable performance indicates that the use of machine learning for HAPI prediction, in the specific clinical setting featured in this study, is not optimal given the utilized variables and available retrospective data. This conclusion is not surprising because predictors that vary linearly and continuously with the outcome are better approximated by a line, not the step-function form that tree-based classification algorithms (42), optimized in machine learning, support. In this context, the selection of the features by expert opinion and testing univariable associations with HAPI outcomes may have biased the selection of our variables to those that vary linearly with HAPI risk.

Previous studies have reported the training and utilization of machine learning models without consulting traditional statistical approaches (14). We find the allure of and immediate acceptance of automated machine learning approaches, especially when done without any assessment of the appropriateness of the approach, a cause for concern due to the implications of how it arrives at its decision. From our study, we reported discordance between some of the predictors found important by the Logistic Regression and machine learning-based modeling approaches. Discordance between model findings reflect differences in model assumptions on the underlying data generating mechanism. In addition, the capability of each model is dependent on how well its assumptions capture the natural mechanisms involved. In this study, the Logistic Regression model does not model interactions, whereas the less parametric machine learning methods (e.g., Random Forest) implicitly model interactions (e.g., presence of a conditional effect) and non-linear associations, which can impact the importance of the main effect (42). Additionally, changes in feature importance between predictors in the presence of collinearity has been well studied. Nonetheless, dealing with these disagreements is part of the challenge of comparing different methodologies.

These differences may potentially confuse the clinician as to which model-learned factors to focus on. For instance, the clinician may focus on records of low friction, average mobility, and NPO status if utilizing either the machine learning or Logistic Regression modeling approaches. However, they may choose to disregard indicators of low nutrition, activity and high moisture while prioritizing smoking status if opting to utilize the machine learning models over Logistic Regression more often (43, 44). Shifting the physician's attention to these machine learning derived predictors may have unintended consequences for the patient. Therefore, it is imperative to resolve any additional uncertainty introduced by these machine learning techniques before seeking to adopt them. Adoption of the machine learning models should be done in concert with the practicing clinician and the prevailing literature. While we presented the results from many models in this work, ultimately it is the domain expert's decision to select from the best performing models an approach whose selected variables best match their clinical perspective. In clinical practice, these models can best be utilized as a “safety check” to catch any missed signs and symptoms after the primary assessment by the clinician (45, 46).

In concert with cautionary advice on machine learning implementations, Logistic Regression approaches are more intuitive, easier to understand and currently more readily adoptable in the biomedical community. The results corroborate with existing literature suggesting that machine learning models are frequently unable to outperform Logistic Regression models in a structured clinical setting (in which relatively few but accurately measured variables are available) (i.e., *all that glitters is not gold*), although a few other studies have disputed this claim (47–49). In general, a modeling approach should be selected which matches the data generating mechanism and agrees with clinical intuition. The machine learning models in this study disregarded important predictors, such as nutrition and activity, both of which were corroborated by evidence from prior studies and through consulting with our medical expert. Since these machine learning models also were unable to outperform the traditional statistical modeling, it would be a safer option to continue to use the Logistic Regression approach in this specific research setting. Nevertheless, in light of recent studies indicating relationships between excluded biomarkers such as albumin and C-reactive protein levels (CRP) (50) in the pressure injury setting, having time-stamped data with access to complete biomarker data may warrant us to revisit our modeling approach to incorporate the agility of machine learning techniques to specify and explore interactions.

In addition, many clinical stakeholders are excited to adopt machine learning technologies. It is worth noting that many academics consider Logistic Regression a machine learning methodology. Regardless of how Logistic Regression is perceived—either as a traditional statistical model or machine learning technique—more important is the performance of the model. Based on the study findings, stakeholders may be willing to incorporate these algorithms into clinical practice to improve healthcare and patient outcomes (e.g., prolonged hospitalization, healthcare expenditures, and patient co-payments).

While SHAP coefficients for the Logistic Regression model converge on the global Logistic Regression model coefficients, they provide a quick and intuitive means for obtaining the patient's risk and how certain predictors contribute to that risk. We further highlight a key difference between SHAP model coefficients and the Logistic Regression coefficients: Logistic Regression beta coefficients are a global descriptor of training set predictors, while SHAP models are fit on held-out test data and can converge to these coefficients. SHAP is useful for generating explanations for a machine learning model to capture heterogeneity in the population by fitting separate models for each individual. While SHAP may be less useful for generating interpretations for the linear model, the software offered to produce these patient-level explanations can be easily deployed into an EMR system for clinical use.

### Limitations

There are a few limitations to our study. The study data was collected from a single institution and our patient demographic (97% white) does not correspond to that across the United States (51). Also, we are unaware of the effect that Dartmouth Hitchcock specific HAPI intervention programs may serve to bias HAPI results (1). While the reported incidence is less than that reported by the National Database of Nursing Quality Indicators (NDNQI; quality surveillance data) and previous cross-sectional surveys (52–54), this may be reflective of specific reductions in HAPI incidence over time potentially related to the HAPI intervention program (1) and more stringent self-validation measures for HAPI identification (8). These inclusion criteria could have led to more accurate identification of the outcome and thus prove beneficial or at least not substantially impact the valid comparison of machine learning techniques. Thus, our results may not generalize to other institutions. It is beyond the scope of this work to explore HAPI predictions outside the hospital setting; although a significant number of pressure injuries occur in long term care facilities, we should be careful to extend conclusions to those patients.

In addition, we were unable to capture all possible clinical covariates or fully utilize real-time repeated measures for this study. The mean length of stay (LOS) for a patient in our study population who does not experience a HAPI is 8.2 days (SD = 9.7) and for those who do experience a HAPI is 30.6 days (SD = 28.6). A short length of stay for a HAPI patient may make it difficult to collect enough repeated measurements (at least 3) to make real-time predictions. Since early-stage pressure injuries are often overlooked, a reduced observation time may limit our ability to make substantial inferences based on sparse information. A real-time predictive model should account for the impact that the length of stay can have on pressure injury incidence while avoiding associated issues with inference, such as record completeness and endogeneity. Nevertheless, the addition of repeated lab measurements, unstructured clinical note data, and modalities such as biomedical imaging and sensor data from wearable technology (55–57), would be advantageous toward developing more sophisticated and actionable real-time predictive models using all information known up until that point. EMR information can be noisy and incomplete in many cases. Adopting noise generating techniques can further regularize these machine learning models to improve robustness to unseen data.

The use of Shapley feature attributions presents a great opportunity to develop a set of explanatory tools to more quickly assess machine learning predictions for any patient outcome. In this study, we used them as a means of comparison to understand which predictors were found to be important for each machine learning model in predicting pressure injuries. The preliminary inspection of these SHAP scores (misalignment between machine learning predictor importance and Logistic Regression coefficients) alerted us to the possibility that the machine learning approaches could potentially mislead the clinician in their treatment of symptoms associated with the occurrence of pressure injuries. Qualitative comparisons between SHAP and other interpretation approaches such as LIME also demonstrated differences in some cases. SHAP is preferred over LIME because: (1) SHAP has been well formulated for tree-based interpretations, (2) improvements in mathematical guarantees over LIME have been well documented, (3) SHAP provides global feature importances, and (4) reports salient interactions. However, this does not necessarily indicate SHAP as the “go to” feature importance technique; discussion of display outputs from other feature importance approaches (e.g., counterfactual explanations; DiCE, Anchor) in collaboration with the clinical domain expert is important when making a final selection (58–62). While the ultimate utility in using SHAP lies in the ability to fit explanatory models for each individual in the case that machine learning approaches dominate, SHAP, in any model application, can generate instance-wise importance values for useful, patient-specific readouts for the clinician.

However, explaining the output of models using SHAP carries several limitations, namely that SHAP has a limited causal interpretation (see Section “Extended Discussion on SHAP Limitations” in Supplementary Material) (63). Several studies have also demonstrated that SHAP can be difficult to interpret in the real-world setting (i.e., to accomplish the goal of compelling a clinician to make a beneficial change based on the model output), in part because: (1) they may underappreciate the stakeholder's “autonomy, dignity and personhood” (i.e., the ability to make a decision or select amongst a set of necessary alternatives), (2) may not appropriately educate stakeholders on how to form a critical, nuanced interpretation of findings, and (3) does not facilitate debate on whether such findings are justified (64–66). Explanations should also consider the viewpoints of all potential project stakeholders, even those not included in algorithmic design and validation. Accordingly, some argue that uncritical acceptance of SHAP model interpretations may disregard crucial input from stakeholders who may lie outside of the direct team of researchers and user testers (e.g., minority patient populations outside of the researchers' target demographics who may interpret from a different perspective or be significantly impacted by findings). Designing EMR interfaces which consider a pluralism of explanations (e.g., assigning greater uncertainty to correlated features or incorporating Bayesian methods) in a less familiar form (e.g., different icons to accentuate less relevant variables and disrupt automatic thinking) may invite critical interpretation of the model findings while remaining sensitive to individuals who may be disempowered in the algorithmic design and interpretation phase (67–70). We point the reader to a few cited studies for those seeking a deeper understanding (63–65, 71–73).

These discussion points should be placed in the context of algorithmic bias and ethical concerns, which can place undue risk on both the clinician stakeholder and underrepresented patient subgroups. For instance, several previous medical AI studies have identified patient demographic features such as self-reported race as potential model confounders (74). In other cases, they may underdiagnose historically underserved races at a higher rate (one popular example is skin lesion image classifiers which screen for Melanoma) (75–79). These confounders and effect modifiers are often unaccounted for when developing and validating machine learning approaches, hampering generalizability. In this study, it is possible that HAPI incidence may be higher for some of these patient subgroups as pointed out by previous literature, but as HAPI incidence is rare and for those that have HAPI, only a small fraction is non-white (Supplementary Table 5), risk scores for other races/ethnicities may be uninformative and non-specific. Such challenges are often exacerbated in rural healthcare settings, which could point to broader multicenter collaborations which could improve the applicability of study findings to underrepresented groups.

## Conclusions

In this study, we demonstrated that a Logistic Regression modeling approach performed comparably to 11 other machine learning methods for HAPI prediction while improving on existing HAPI prediction benchmarks. In addition, we highlight the potential to integrate patient-level explanations into existing EMR systems. We believe that future applications of machine learning algorithms, in conjunction with traditional statistical models, that utilize repeated measurements, laboratory markers and unstructured clinical notes will provide a promising opportunity to build real-time prediction mechanisms that can be readily embedded into an EMR system to alert clinical staff to high-risk patients.

## Data Availability Statement

The datasets presented in this article are not readily available because of participant privacy concerns. Requests to access the datasets should be directed to joshua.j.levy@dartmouth.edu.

## Ethics Statement

The studies involving human participants were reviewed and approved by DH Human Research Protection Program (IRB). Written informed consent for participation was not required for this study in accordance with the national legislation and the institutional requirements.

## Author Contributions

RE, GF, and MM conceived of study. JFL conducted initial analyses and had written the initial manuscript draft. JJL provided project oversight, mentorship, and had written subsequent manuscript drafts. All authors contributed to manuscript writing and editing. All authors contributed to the article and approved the submitted version.

## Funding

The Dartmouth Clinical and Translational Science Institute supported RTE under the Award Number UL1TR001086 from the National Center for Advancing Translational Sciences (NCATS) of the National Institutes of Health (NIH). JJL was supported by the Burroughs Wellcome Fund Big Data in the Life Sciences training grant at Dartmouth and NIH grant P20GM104416 subaward.

## Conflict of Interest

The authors declare that the research was conducted in the absence of any commercial or financial relationships that could be construed as a potential conflict of interest.

## Publisher's Note

All claims expressed in this article are solely those of the authors and do not necessarily represent those of their affiliated organizations, or those of the publisher, the editors and the reviewers. Any product that may be evaluated in this article, or claim that may be made by its manufacturer, is not guaranteed or endorsed by the publisher.

## References

[1] Miller MW Emeny RT Freed GL. Reduction of hospital-acquired pressure injuries using a multidisciplinary team approach: a descriptive study. Wounds. (2019) 31:108–13. 30802207 PMC6586476

[2] Thomas DR. Does pressure cause pressure ulcers? An inquiry into the etiology of pressure ulcers. J Am Med Direct Assoc. (2010) 11:397–405. 10.1016/j.jamda.2010.03.007 20627180

[3] Epidemiology Pathogenesis and and Risk Assessment of Pressure-Induced Skin and Soft Tissue Injury - UpToDate. Available online at: https://www.uptodate.com/contents/epidemiology-pathogenesis-and-risk-assessment-of-pressure-induced-skin-and-soft-tissue-injury?search=Epidemiology,%20pathogenesis,%20and%20risk%20assessment%20of%20pressure-induced%20skin%20and%20soft%20tissue%20injury&source=search_result&selectedTitle=1~150&usage_type=default&display_rank=1 (accessed November 26, 2019).

[4] Padula WV Pronovost PJ Makic MBF Wald HL Moran D Mishra MK. Value of hospital resources for effective pressure injury prevention: a cost-effectiveness analysis. BMJ Qual Saf. (2019) 28:132–41. 10.1136/bmjqs-2017-007505 30097490 PMC6365919

[5] Coomer NM Kandilov AMG. Impact of hospital-acquired conditions on financial liabilities for Medicare patients. Am J Infect Control. (2016) 44:1326–34. 10.1016/j.ajic.2016.03.025 27174461

[6] Hanley JA McNeil BJ. The meaning and use of the area under a receiver operating characteristic (ROC) curve. Radiology. (1982) 143:29–36. 10.1148/radiology.143.1.7063747 7063747

[7] Chen H-L Shen W-Q Liu P. A meta-analysis to evaluate the predictive validity of the braden scale for pressure ulcer risk assessment in long-term care. Ostomy Wound Manage. (2016) 62:20–8. 27668477

[8] Miller MW Emeny RT Snide JA Freed GL. Patient-specific factors associated with pressure injuries revealed by electronic health record analyses. Health Inform J. (2019) 26:474-485. 10.1177/1460458219832053 30880544 PMC6751028

[9] Hyun S Vermillion B Newton C Fall M Li X Kaewprag P. Predictive validity of the Braden scale for patients in intensive care units. Am J Crit Care. (2013) 22:514–20. 10.4037/ajcc2013991 24186823 PMC4042540

[10] Kanevsky J Corban J Gaster RS Kanevsky A Lin SJ Gilardino MS. Big data and machine learning in plastic surgery: a new frontier in surgical innovation. Plast Reconst Surg. (2016) 137:890e−7e. 10.1097/PRS.0000000000002088 27119951

[11] Kaewprag P Newton C Vermillion B Hyun S Huang K Machiraju R. Predictive modeling for pressure ulcers from intensive care unit electronic health records. AMIA Jt Summits Transl Sci Proc. (2015) 2015:82–6. 26306245 PMC4525237

[12] Nakamura Y Ghaibeh AA Setoguchi Y Mitani K Abe Y Hashimoto I. On-admission pressure ulcer prediction using the nursing needs score. JMIR Med Inform. (2015) 3:e8. 10.2196/medinform.3850 25673118 PMC4342622

[13] Kaewprag P Newton C Vermillion B Hyun S Huang K Machiraju R. Predictive models for pressure ulcers from intensive care unit electronic health records using Bayesian networks. BMC Med Inform Decis Mak. (2017) 17:65. 10.1186/s12911-017-0471-z 28699545 PMC5506589

[14] Alderden J Pepper GA Wilson A Whitney JD Richardson S Butcher R. Predicting pressure injury in critical care patients: a machine-learning model. Am J Crit Care. (2018) 27:461–8. 10.4037/ajcc2018525 30385537 PMC6247790

[15] Hyun S Moffatt-Bruce S Cooper C Hixon B Kaewprag P. Prediction model for hospital-acquired pressure ulcer development: retrospective cohort study. JMIR Med Inform. (2019) 7:e13785. 10.2196/13785 31322127 PMC6670273

[16] Ravi V Zheng J Subramaniam A Thomas LG Showalter J Frownfelter J. Artificial Intelligence (AI) and machine learning (ML) in risk prediction of hospital acquired pressure injuries (HAPIs) among oncology inpatients. JCO. (2019) 37:e18095. 10.1200/JCO.2019.37.15_suppl.e18095

[17] Cramer EM Seneviratne MG Sharifi H Ozturk A Hernandez-Boussard T. Predicting the incidence of pressure ulcers in the intensive care unit using machine learning. eGEMs (Generat Evid Methods Improve Patient Outcomes). (2019) 7:49. 10.5334/egems.307 31534981 PMC6729106

[18] Zrelak PA Utter GH Tancredi DJ Mayer LG Cerese J Cuny J. How accurate is the AHRQ patient safety indicator for hospital-acquired pressure ulcer in a national sample of records? J Healthcare Qual. (2015) 37:287–97. 10.1111/jhq.12052 24118246

[19] Azur MJ Stuart EA Frangakis C Leaf PJ. Multiple imputation by chained equations: what is it and how does it work? Int J Methods Psychiatr Res. (2011) 20:40–9. 10.1002/mpr.329 21499542 PMC3074241

[20] Rennie JDM Shih L Teevan J Karger DR. Tackling the poor assumptions of naive bayes text classifiers. In: Proceedings of the Twentieth International Conference on International Conference on Machine Learning. Washington, DC: AAAI Press (2003). p. 616–23.

[21] Quinlan JR. Induction of decision trees. Mach Learn. (1986) 1:81–106. 10.1007/BF00116251

[22] Ho TK. Random decision forests. In: Proceedings of the Third International Conference on Document Analysis and Recognition. (Volume 1). IEEE Computer Society, Washington, DC (1995). p. 278.

[23] Chen T Guestrin C. XGBoost: a scalable tree boosting system. In: Proceedings of the 22Nd ACM SIGKDD International Conference on Knowledge Discovery and Data Mining. New York, NY: ACM (2016). p. 785–794.

[24] Kleinbaum DG Klein M. Introduction to logistic regression. In: Kleinbaum DG Klein M editors. Logistic Regression: A Self-Learning Text. New York, NY: Springer (2010). p. 1–39.

[25] Lachenbruch PA Sneeringer C Revo LT. Robustness of the linear and quadratic discriminant function to certain types of non-normality. Commun Stat. (1973) 1:39–56. 10.1080/03610927308827006

[26] LeCun Y Bengio Y Hinton G. Deep learning. Nature. (2015) 521:436–44. 10.1038/nature14539 26017442

[27] Hearst M Dumais ST Osman E Platt J Scholkopf B. Support vector machines. Intelligent systems and their applications. IEEE. (1998) 13:18–28. 10.1109/5254.708428

[28] Cover T Hart P. Nearest neighbor pattern classification. IEEE Trans Inform Theory. (1967) 13:21–7. 10.1109/TIT.1967.1053964

[29] Chipman HA George EI McCulloch RE. BART Bayesian additive regression trees. Ann Appl Stat. (2010) 4:266–98. 10.1214/09-AOAS28531460678

[30] Pandis N. Logistic regression: part 1. Am J Orthod Dentofacial Orthoped. (2017) 151:824–5. 10.1016/j.ajodo.2017.01.017 28364908

[31] Biau G. Analysis of a random forests model. J Mach Learn Res. (2012) 13:1063–95. 10.5555/2188385.2343682

[32] Longadge R Dongre S. Class imbalance problem in data mining review. arXiv [Preprint]. (2013). arXiv: 1305.17071. Available online at: https://arxiv.org/abs/1305.1707v1

[33] Fernandez A Garcia S Herrera F Chawla NV. SMOTE for learning from imbalanced data: progress and challenges, marking the 15-year anniversary. J Artificial Intellig Res. (2018) 61:863–905. 10.1613/jair.1.11192

[34] Bathaee Y. The Artificial Intelligence Black Box and the Failure of Intent and Causation Cambridge, MA: Harvard Journal of Law & Technology (2018).

[35] Lundberg SM Lee S-I. A unified approach to interpreting model predictions. In: Guyon I Luxburg UV Bengio S Wallach H Fergus R Vishwanathan S., editors. Advances in Neural Information Processing Systems, 30. Red Hook, NY: Curran Associates, Inc. (2017). p. 4765–74.

[36] Webber W Moffat A Zobel J. A similarity measure for indefinite rankings. ACM Trans Inf Syst. (2010) 28:20:1–20:38. 10.1145/1852102.1852106

[37] Ribeiro MT Singh S Guestrin C. “Why should i trust you?”: explaining the predictions of any classifier. In: *Proceedings of the 22nd ACM SIGKDD International Conference on Knowledge Discovery and Data Mining*. New York, NY: Association for Computing Machinery (2016). p. 1135–44.

[38] Ribeiro MT Singh S Guestrin C. Anchors: high-precision model-agnostic explanations. In: Proceedings of the AAAI Conference on Artificial Intelligence. New Orleans, Louisiana (2018). p. 32.

[39] Mothilal RK Sharma A Tan C. Explaining machine learning classifiers through diverse counterfactual explanations. In: Proceedings of the 2020 Conference on Fairness, Accountability, and Transparency. New York, NY: Association for Computing Machinery (2020). p. 607–17.

[40] Molnar C. Interpretable Machine Learning: A Guide For Making Black Box Models Explainable. (2022).

[41] Salvatier J Wiecki TV Fonnesbeck C. Probabilistic programming in Python using PyMC3. PeerJ Computer Science. (2016) 2:e55. 10.7717/peerj-cs.55 PMC10495961 37705656

[42] Levy JJ O'Malley AJ. Don't dismiss logistic regression: the case for sensible extraction of interactions in the era of machine learning. BMC Med Res Methodol. (2020) 20:171. 10.1186/s12874-020-01046-3 32600277 PMC7325087

[43] Alderden J Rondinelli J Pepper G Cummins M Whitney J. Risk factors for pressure injuries among critical care patients: a systematic review. Int J Nurs Stud. (2017) 71:97–114. 10.1016/j.ijnurstu.2017.03.012 28384533 PMC5485873

[44] Saghaleini SH Dehghan K Shadvar K Sanaie S Mahmoodpoor A Ostadi Z. Pressure ulcer and nutrition. Indian J Crit Care Med. (2018) 22:283–9. 10.4103/ijccm.IJCCM_277_17 29743767 PMC5930532

[45] Gerke S Minssen T Cohen G. Ethical and legal challenges of artificial intelligence-driven healthcare. Artif Intellig Healthcare. (2020) 12:295–336. 10.1016/B978-0-12-818438-7.00012-532245804

[46] Rigby MJ. Ethical dimensions of using artificial intelligence in health care. AMA J Ethics. (2019) 21:121–4. 10.1001/amajethics.2019.12134346319

[47] Couronné R Probst P Boulesteix A-L. Random forest versus logistic regression: a large-scale benchmark experiment. BMC Bioinformatics. (2018) 19:270. 10.1186/s12859-018-2264-5 30016950 PMC6050737

[48] Christodoulou E Ma J Collins GS Steyerberg EW Verbakel JY Van Calster B. systematic review shows no performance benefit of machine learning over logistic regression for clinical prediction models. J Clin Epidemiol. (2019) 110:12–22. 10.1016/j.jclinepi.2019.02.004 30763612

[49] Kirasich K Smith T Sadler B. Random Forest vs Logistic Regression: Binary Classification for Heterogeneous Datasets. SMU Data Sci Rev. (2018). Available online at: https://scholar.smu.edu/datasciencereview/vol1/iss3/9

[50] Sugino H Hashimoto I Tanaka Y Ishida S Abe Y Nakanishi H. Relation between the serum albumin level and nutrition supply in patients with pressure ulcers: retrospective study in an acute care setting. J Med Investig. (2014) 61:15–21. 10.2152/jmi.61.15 24705743

[51] Li Y Yin J Cai X Temkin-Greener H Mukamel DB. Association of race and sites of care with pressure ulcers in high-risk nursing home residents. JAMA. (2011) 306:179–86. 10.1001/jama.2011.942 21750295 PMC4108174

[52] Bergquist-Beringer S Dong L He J Dunton N. Pressure ulcers and prevention among acute care hospitals in the united states. JCJQPS. (2013) 39:404–14. 10.1016/S1553-7250(13)39054-0 24147352

[53] Kayser SA VanGilder CA Lachenbruch C. Predictors of superficial and severe hospital-acquired pressure injuries: a cross-sectional study using the International Pressure Ulcer Prevalence^TM^ survey. Int J Nurs Stud. (2019) 89:46–52. 10.1016/j.ijnurstu.2018.09.003 30339955

[54] Díaz-Caro I García Gómez-Heras S. Incidence of hospital-acquired pressure ulcers in patients with “minimal risk” according to the “Norton-MI” scale. PLoS ONE. (2020) 15:e0227052. 10.1371/journal.pone.0227052 31914154 PMC6948734

[55] Cicceri G De Vita F Bruneo D Merlino G Puliafito A. A deep learning approach for pressure ulcer prevention using wearable computing. Human-centric Comput Inform Sci. (2020) 10:5. 10.1186/s13673-020-0211-8

[56] Elmogy M Zapirain B Burns C Elmaghraby A El-Baz A. Tissues Classification for pressure ulcer images based on 3D convolutional neural network. In: 2018 25th IEEE International Conference on Image Processing. Athens: IEEE (2018). p. 3139–43.

[57] Fergus P Chalmers C Tully D. Collaborative pressure ulcer prevention: an automated skin damage and pressure ulcer assessment tool for nursing professionals, patients, family members and carers. arXiv:1808.06503 (2018). 10.48550/arXiv.1808.06503

[58] ElShawi R Sherif Y Al-Mallah M Sakr S. Interpretability in healthcare: a comparative study of local machine learning interpretability techniques. Comput Intellig. (2021) 37:1633–50. 10.1111/coin.12410

[59] Stiglic G Kocbek P Fijacko N Zitnik M Verbert K Cilar L. Interpretability of machine learning-based prediction models in healthcare. WIREs Data Mining Knowl Discov. (2020) 10:e1379. 10.1002/widm.1379

[60] Belle V Papantonis I. Principles and practice of explainable machine learning. Front Big Data. (2021) 4:688969. 10.3389/fdata.2021.688969 34278297 PMC8281957

[61] Jesus S Belém C Balayan V Bento J Saleiro P Bizarro P. How can I choose an explainer? An application-grounded evaluation of *post-hoc* explanations. In: Proceedings of the 2021 ACM Conference on Fairness, Accountability, and Transparency. New York, NY: Association for Computing Machinery (2021). p. 805–15.

[62] Watson DS Gultchin L Taly A Floridi L. Local explanations via necessity and sufficiency: unifying theory and practice. In: Proceedings of the Thirty-Seventh Conference on Uncertainty in Artificial Intelligence. PMLR, Virtual. (2021). p. 1382–92.

[63] Ma S Tourani R. Predictive and causal implications of using shapley value for model interpretation. In: Proceedings of the 2020 KDD Workshop on Causal Discovery. PMLR, San Diego, CA. (2020). p. 23–38.

[64] Kumar IE Venkatasubramanian S Scheidegger C Friedler S. Problems with Shapley-value-based explanations as feature importance measures. In: Proceedings of the 37th International Conference on Machine Learning. PMLR, Virtual. (2020). p. 5491–500.

[65] Hancox-Li L Kumar IE. Epistemic values in feature importance methods: lessons from feminist epistemology. In: Proceedings of the 2021 ACM Conference on Fairness, Accountability, and Transparency. Toronto, ON. (2021). p. 817–26.

[66] Schwab P Karlen W. CXPlain: causal explanations for model interpretation under uncertainty. In: Proceedings of the 33rd International Conference on Neural Information Processing Systems. Red Hook, NY: Curran Associates Inc (2019). p. 10220–30.

[67] Zhao X Huang W Huang X Robu V Flynn D. BayLIME: Bayesian local interpretable model-agnostic explanations. In: Proceedings of the Thirty-Seventh Conference on Uncertainty in Artificial Intelligence. PMLR. Virtual Event. (2021). p. 887–96.

[68] Shaikhina T Bhatt U Zhang R Georgatzis K Xiang A Weller A. Effects of uncertainty on the quality of feature importance explanations. In: AAAI Workshop on Explainable Agency in Artificial Intelligence. AAAI Press (2021).

[69] Slack D Hilgard A Singh S Lakkaraju H. Reliable *post hoc* explanations: modeling uncertainty in explainability. In: Advances in Neural Information Processing Systems. Curran Associates, Inc.(2021) 34. p. 9391–404. Available online at: https://proceedings.neurips.cc/paper/2021/hash/4e246a381baf2ce038b3b0f82c7d6fb4-Abstract.html

[70] Li X Zhou Y Dvornek NC Gu Y Ventola P Duncan JS. Efficient Shapley explanation for features importance estimation under uncertainty. In: International Conference on Medical Image Computing and Computer-Assisted Intervention. Lima, Peru: Springer (2020). p. 792–801. 10.1007/978-3-030-59710-8_77 PMC8299327 34308439

[71] Janzing D Minorics L Blöbaum P. Feature relevance quantification in explainable AI: a causal problem. In: International Conference on Artificial Intelligence and Statistics. PMLR, Virtual Event. (2020). p. 2907–16.

[72] Dai J Upadhyay S Bach SH Lakkaraju H. What will it take to generate fairness-preserving explanations? arXiv preprint arXiv:2106.13346 (2021). 10.48550/arXiv.2106.13346

[73] Barocas S Selbst AD Raghavan M. The hidden assumptions behind counterfactual explanations and principal reasons. In: Proceedings of the 2020 Conference on Fairness, Accountability, and Transparency. Barcelona, Spain. (2020). p. 80–9.

[74] Gichoya JW Banerjee I Bhimireddy AR Burns JL Celi LA Chen L-C. AI recognition of patient race in medical imaging: a modelling study. Lancet Digital Health. (2022) 4:e406–14. 10.1016/S2589-7500(22)00063-2 35568690 PMC9650160

[75] Guo LN Lee MS Kassamali B Mita C Nambudiri VE. Bias in, bias out: underreporting and underrepresentation of diverse skin types in machine learning research for skin cancer detection–a scoping review. J Am Acad Dermatol. (2021). 10.1016/j.jaad.2021.06.884 34252465

[76] Zakhem GA Fakhoury JW Motosko CC Ho RS. Characterizing the role of dermatologists in developing artificial intelligence for assessment of skin cancer. J Am Acad Dermatol. (2021) 85:1544–56. 10.1016/j.jaad.2020.01.028 31972254

[77] Char DS Abràmoff MD Feudtner C. Identifying ethical considerations for machine learning healthcare applications. Am J Bioeth. (2020) 20:7–17. 10.1080/15265161.2020.1819469 33103967 PMC7737650

[78] Char DS Shah NH Magnus D. Implementing machine learning in health care—addressing ethical challenges. N Engl J Med. (2018) 378:981. 10.1056/NEJMp1714229 29539284 PMC5962261

[79] McCradden MD Joshi S Mazwi M Anderson JA. Ethical limitations of algorithmic fairness solutions in health care machine learning. Lancet Digital Health. (2020) 2:e221–3. 10.1016/S2589-7500(20)30065-0 33328054

