# Supplementary material for: Machine Learning Approaches for Hospital Acquired Pressure Injuries: A Retrospective Study of Electronic Medical Records
Source: Front Med Technol. 2022 Jun 16;4:926667. doi: 10.3389/fmedt.2022.926667 (PMC9243224; doi:10.3389/fmedt.2022.926667)
Supplement: Supplementary file 1 [file Data_Sheet_1.pdf]

Supplementary Materials

Supplementary Table 1: Previous HAPI prediction studies– brief description, final modeling approach selected with corresponding C-Statistic

Study	Description	Final Approach	Final C-Statistic
**Miller et al., 2019**	n/a	Braden Scale	0.67
**Hyun et al., 2013**	n/a	Braden Scale	0.77
**Kaewprag et al., 2015**	Six machine learning models on ICU patients; Braden score, medications, comorbidities	Logistic Regression	0.83
**Nakamura et al., 2015**	General population; Nurse Needs Score	Logistic Regression	0.84
**Kaewprag et al., 2017**	Predictors similar to Kaewprag et al., 2015	Bayesian Network	0.83
**Aldermen et al., 2018**	No methods comparison	Random Forest	0.79
**Hyun et al., 2019**	ICU specific features	Logistic Regression	0.737
**Ravi et al., 2019**	Oncology patients	Jvion CCSM AI Platform	0.84
**Cramer et al., 2019**	Six machine learning models	Logistic Regression	n/a
**Current Study**	Twelve machine learning models; largest cohort as compared to other studies	Logistic Regression	0.91

Supplementary Table 2: Percent breakdown of stage amongst HAPI cases reviewed from nursing records via an event reporting system (data from Miller et. al., 2019); 2015-2018

Year	*2015*				*2016*			
	1Q	2Q	3Q	4Q	1Q	2Q	3Q	4Q
HAPI Stage								
Unstageable	6	8	2	5	5	1	0	1
Suspected Deep Tissue Injury	17	26	26	18	30	14	12	16
Stage 3	6	1	1	0	0	0	0	0
Stage 2	8	12	9	6	12	6	10	2
Stage 1	3	9	6	7	6	4	3	5

Year	*2017*				*2018*		*2015-2018*	
	1Q	2Q	3Q	4Q	1Q		Total	Proportion
HAPI Stage								
Unstageable	6	8	2	5	5		31	7.2%
Suspected Deep Tissue Injury	17	26	26	18	30		233	54.1%
Stage 3	6	1	1	0	0		8	1.9%
Stage 2	8	12	9	6	12		96	22.3%
Stage 1	3	9	6	7	6		63	14.6%

Supplementary Table 3: Percent breakdown of whether HAPI was medical device related via an event reporting system (data from Miller et. al., 2019); 2nd Quarter 2017-2nd Quarter 2018

Medical Device Related?	*Total*	*Proportion*
Yes	34	36.2%
No	55	58.5%
Unknown	5	5.3%

Supplementary Table 4: Dictionary of utilized predictors and variable names in models

Predictor	Variable Name	Description
Age	Ageatarrival	Patient age when they arrived at the hospital
Sex	male	Indicator variable equal to 1 if the patient is male and 0 if the patient is female
Race	white	Indicator variable equal to 1 if the patient has declared their race as white/Caucasian and 0 if they have not declared a race of white/Caucasian
Smoking history	hxsmoke	Indicator variable equal to 1 if the patient has a history of smoking cigarettes and 0 if the patient has no history of smoking cigarettes.
Body Mass Index	bmitri	Ordinal variable indicating if the patient was “normal” (18.5 <= x < 34.9, “underweight” (< 18.5) or “overweight” (>= 35) according to the study cut-points.
Admitted to ICU	admit_icu	Indicator variable equal to 1 if the patient has spent any time in the ICU and 0 otherwise
Ambulation status	ambulate	Indicator variable whether the patient had mobility orders indicating that the patient should ambulate at any point during the admission
Bed rest orders	bedrest	A variable indicating whether the patient had mobility orders indicating that the patient should be restricted to bed rest at any point during the admission
Nothing by mouth	npo	A variable indicating whether the patient had diet orders indicating that the patient should take nothing by mouth at any point during the admission

Time in OR	timeinor	The amount of time (in days) the patient spent in the OR this admission
Malnutrition	malnut	A variable indicating whether the patient had a diagnosis of malnutrition on this admission
Charlson Score	charlsonscore	Total score based on Charlson comorbidity index
Mean Braden Score	avgbraden	Average total Braden score for this admission
Minimum Braden Score	lowbraden	Lowest total Braden score for this admission
Mean moisture	avgmois	Average score (1-4) for the degree to which the skin is exposed to moisture on the Braden Scale
Mean sensory perception	avgsensperc	Average score (1-4) for the patient's ability to respond meaningfully to pressure related discomfort on the Braden Scale
Mean activity	avgact	Average score (1-4) for the degree of physical activity on the Braden Scale
Mean mobility	avgmob	Average score (1-4) for the patient's ability to change and control body position on the Braden Scale
Mean nutrition	avgnut	Average score (1-4) for the patient's usual food intake patterns on the Braden Scale
Mean friction	avgfric	Average score (1-3) for the level of friction and shear when moving patient on the Braden Scale
Mean moisture	avgmois	Average score (1-4) for the degree to which the skin is exposed to moisture on the Braden Scale
Minimum moisture	lowmois	Lowest score (1-4) for the degree to which the skin is exposed to moisture on the Braden Scale
Minimum sensory perception	lowsensperc	Lowest score (1-4) for the patient's ability to respond meaningfully to pressure related discomfort on the Braden Scale
Minimum activity	lowact	Lowest score (1-4) for the degree of physical activity on the Braden Scale
Minimum mobility	lowmob	Lowest score (1-4) for the patient's ability to change and control body position on the Braden Scale

Minimum nutrition	lownut	Lowest score (1-4) for the patient's usual food intake patterns on the Braden Scale
Minimum friction	lowfric	Lowest score (1-3) for the level of friction and shear when moving patient on the Braden Scale

Supplementary Table 5: Patient Demographics and Braden Scores stratified by HAPI. Two sample t-tests were used for continuous variables and chi-squared tests for categorical variables.

Variable	Missing Values	HAPI Absent	HAPI Present	P value
N		56986	241	
Ageatarrival, mean (SD)	0	60.1 (18.4)	65.5 (15.0)	<0.001
Male, n (%)	0	29536 (51.8)	94 (39.0)	<0.001
Yes		27450 (48.2)	147 (61.0)	
No	0	29536 (51.8)	94 (39.0)	
White, n (%)				0.905
Yes		55618 (97.6)	236 (97.9)	
No	0	1368 (2.4)	5 (2.1)	
Hxsmoke, n (%)				0.271
Yes		28360 (49.8)	129 (53.5)	
No	0	28626 (50.2)	112 (46.5)	
Bmitri, n (%)				0.007
normal	10057	36265 (77.2)	139 (76.4)	
overweight		9233 (19.6)	30 (16.5)	
underweight		1490 (3.2)	13 (7.1)	
Npo, n (%)				<0.001
Yes		37723 (66.2)	224 (92.9)	
No	0	19263 (33.8)	17 (7.1)	
Timeinor, mean (SD)	33443	0.1 (0.1)	0.2 (0.2)	<0.001
Admlt_icu, n (%)				<0.001
Yes		9508 (16.7)	90 (37.3)	
No	0	47478 (83.3)	151 (62.7)	
Ambulate, n (%)				0.974
Yes		11893 (20.9)	50 (20.7)	
No	0	45093 (79.1)	191 (79.3)	
Bedrest, n (%)				<0.001
Yes		16632 (29.2)	121 (50.2)	
No	0	40354 (70.8)	120 (49.8)	
Malnut, n (%)				<0.001
Yes		4482 (7.9)	98 (40.7)	
No	0	52504 (92.1)	143 (59.3)	

Avgmois, mean (SD)	0	3.7 (0.4)	3.3 (0.3)	<0.001
Lowmois, mean (SD)	0	3.2 (0.7)	2.3 (0.8)	<0.001
Avgsensperc, mean (SD)	0	3.6 (0.5)	2.9 (0.7)	<0.001
Lowsensperc, mean (SD)	0	3.1 (0.9)	1.9 (0.9)	<0.001
Avgact, mean (SD)	0	2.7 (0.8)	1.9 (0.7)	<0.001
Lowact, mean (SD)	0	2.0 (1.0)	1.2 (0.5)	<0.001
Avgmob, mean (SD)	0	3.2 (0.6)	2.5 (0.6)	<0.001
Lowmob, mean (SD)	0	2.7 (0.8)	1.7 (0.7)	<0.001
Avgnut, mean (SD)	0	2.8 (0.5)	2.4 (0.4)	<0.001
Lownut, mean (SD)	0	2.2 (0.8)	1.3 (0.6)	<0.001
Avgfric, mean (SD)	0	2.6 (0.4)	2.0 (0.4)	<0.001
Lowfric, mean (SD)	0	2.2 (0.7)	1.2 (0.4)	<0.001
Lowbraden, mean (SD)	0	16.2 (3.5)	11.2 (2.8)	<0.001
Avgbraden, mean (SD)	0	18.5 (2.7)	14.9 (2.4)	<0.001
Charlsonscore, mean (SD)	0	0.5 (1.6)	0.4 (1.5)	0.13

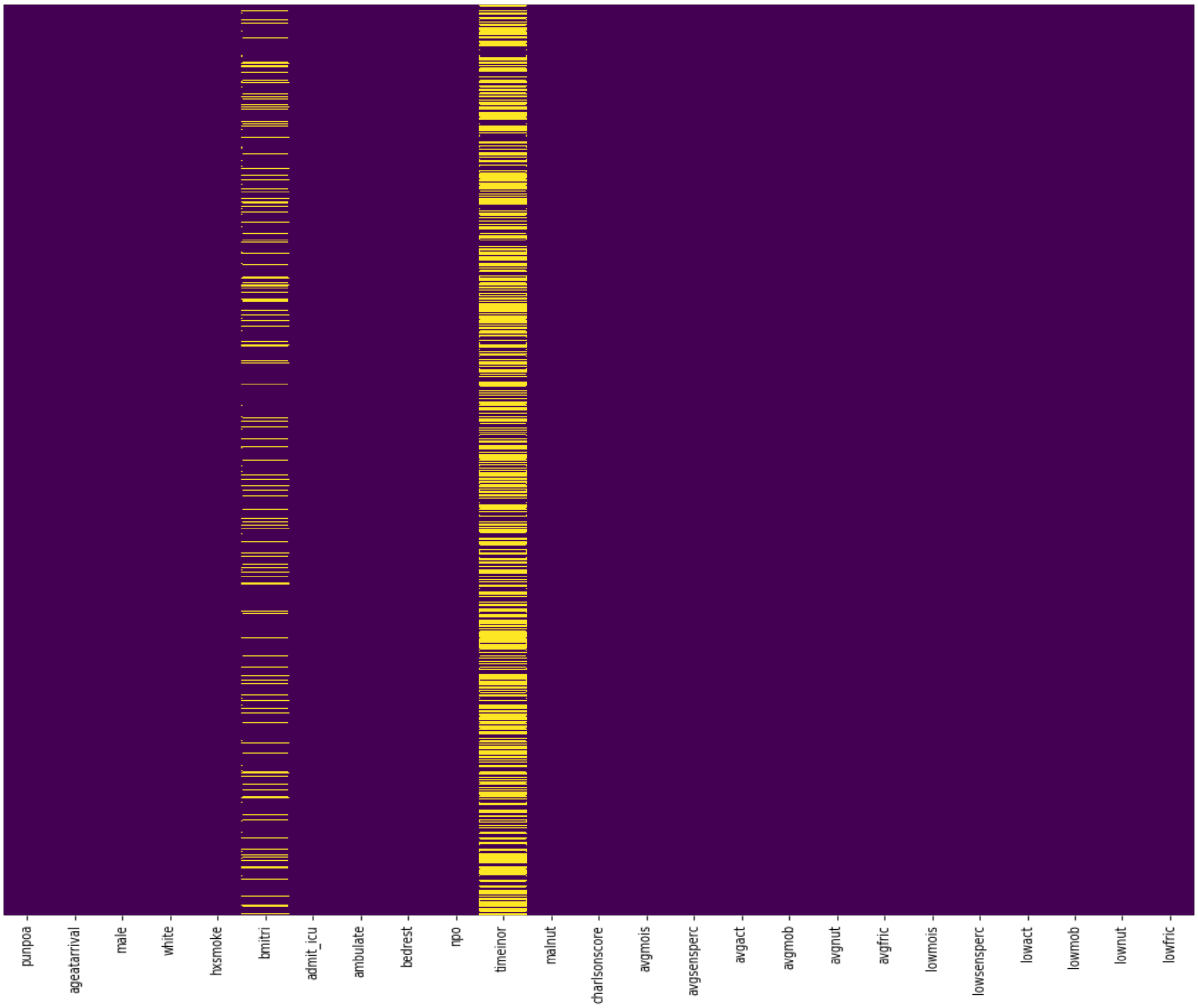

Supplementary Figure 1: Summary of missing values across cohort (BMI and Time in OR)

Further Description of Analytical Approaches

Naive Bayes

Naive Bayes is a simple modeling approach that utilizes Bayes' Theorem to calculate and maximize posterior class probabilities; *maximum a posteriori* (MAP) hypothesis is based on the probability of observing each of the predictors d_i (assumed to be independent), $P(d_i|c)$, given the selection of that class or outcome *c*:

$$\mathrm{MAP} = \max_{c \in C}\left(\Pi_i \frac{P(d_i|c) * P(c)}{P(d_i)}\right)$$

$P(c)$ represents the probability of finding the class in the dataset; observing the outcome more often means it should be predicted as the outcome more often. When $P(d_i|c)$ is high, this means that the observed data is highly probable given the observed outcome and as such, the outcome should be predicted.

Decision Trees

Decision trees are computational heuristics that generate a series of binary splitting rules over the predictors that serve to divide the samples into subgroups. Each subsequent split should more meaningfully inform the prediction of an outcome given the previous split. This criterion is actualized through the measure of information gain, where the entropy, a measure of disorder of labels within bins denoted by the splits (S_1/S_2), should maximally decrease in subsequent splits as compared to the previous splits (S). This is formulated as such:

$$G(S) = H(S) - \left(\frac{n_{S_1}}{n_S} * H(S_1) + \frac{n_{S_2}}{n_S} * H(S_2)\right)$$

Another commonly used criterion is Gini Impurity, which measures how often a randomly chosen element would be incorrectly identified (p_i is the probability of extracting the *ith* label from a split S). The maximal decrease in impurity is used to form decision splits; here, the formula for Gini Impurity is given by:

$$G(S) = 1 - \left(\sum_i p_i^2\right)$$

Random Forest

The random forest approach extends the decision tree method to make predictions based on consensus votes from multiple constructed decision trees. The algorithm operates by first picking a subset of “n” out of a total of “N” variables available. Then it constructs a decision tree using the same methodology as mentioned above (one tree/learner is a collection of decision splits), but alternatively at each split within each tree picking a new n subset of features and bootstrapping the training samples. Each resultant tree/learner makes a HAPI classification and the predictions from each of the trees are tallied to derive the final prediction of the “forest”. This algorithm is considered to be an ensemble approach, where improvements are derived by the incorporation of several weaker models.

Predictor importance is typically assessed using the mean decrease in Gini coefficient (node impurity), where the reduction of Gini impurity is calculated for each variable and averaged across all decision trees where the variable had appeared. However, evaluating the importance of predictors using this criterion may assign lower importance to higher cardinal and/or collinear predictors by spreading importance between predictors that have similar likelihood of being selected at a higher decision node (while the other predictor may be selected at a node with lower impurity decrease, and vice versa across different decision trees). Permutation-based methods (eg. evaluating changes in model performance by removing a predictor and/or in combinations with other predictors) are therefore recommended.

XGBoost

XGBoost is another ensemble method that incorporates decision trees as its base algorithm. Random Forests employ bagging techniques, which reduces the estimate variance by bootstrap aggregation, i.e. averaging multiple model estimates. In contrast, boosting, as utilized by XGBoost, reduces the bias introduced by individual weak learners by sequentially constructing learners in a way that assigns higher weights to misclassified samples from the previous learner. Learners are also weighted based on their ability to classify data when it comes time to aggregate the predictions of the individual learners.

In summary, Random Forest parallelizes the construction and voting of decision trees to arrive at a conclusion, while XGBoost sequentially and greedily updates learners and weights assigned to samples based on mistakes from previous learners.

Logistic Regression

Binary logistic regression, a member of the generalized linear model (GLM) family, is a classic statistical method used to model binary outcomes. GLMs can model response variables with non-normally distributed errors, and can also utilize nonlinear link functions such as the logit function to depict the direct relationship between the expected value of the dependent variable and the independent variables:

$$\mathrm{Logit}(p) = \log\left(\frac{p}{1-p}\right) = \beta_0 + \beta_1 * x_1 + \beta_2 * x_2 + \cdots + \beta_n * x_n$$

Which can be expressed as:

$$P(Y = 1 | x_1, x_2, \ldots, x_n) = \frac{1}{1 + e^{-(\beta_0 + \beta_1 * x_1 + \beta_2 * x_2 + \cdots + \beta_n * x_n)}}$$

Compared to the aforementioned machine learning methods, these models have highly interpretable model coefficients. The exponentiation of summed coefficients corresponds to the odds ratio of the outcome across a unit change in the value of the predictor - a clinically interpretable quantity.

In situations where many potential predictors are available, these models can be regularized using LASSO, Ridge and Elastic Net to home in on important predictors. The coefficients of the LASSO, Ridge and Elastic Net models do not represent valid odds ratios, thus a traditional Logistic Regression model, unregularized (without the use of LASSO, Ridge and Elastic Net) must be utilized to derive valid odds ratios. In this paper, we elected to not penalize the logistic regression model as the number of predictors was relatively small.

Additional Comparison Approaches

While we have provided in-depth descriptions and analysis of several machine learning algorithms commonly employed by the biomedical community, particularly for HAPI prediction, these approaches only represent a small subset of machine learning approaches which could have been utilized. As such, we also tested and compared seven additional machine learning models for this task. For instance, fully Bayesian approaches (e.g., Bayesian Logistic Regression, Bayesian Additive Regression Trees) can limit bias through the selection of weakly informative priors which penalize the model's complexity. We provide brief descriptions of all algorithms used in this study below:

Supplementary Table 6: Brief Description of all modeling approaches

Approach	Brief Description
**Logistic Regression**	• Establishes a linear relationship with a binary outcome on the log-odds scale.<br>• Exponentiated regression coefficients represent odds ratios.

**Naive Bayes**	• Simple application of Bayes' theorem to maximize posterior class probabilities.<br>• Weights the likelihood of observing the predictor with a class-specific prior.
**Decision Trees**	Fits a set of conditional decision splits which maximizes information gain or minimizes impurity.
**Random Forest**	Aggregates the outcome from many independently fit decision trees under the premise that each tree alone exhibits bias for a subset of predictors / influential observations.
**XGBoost**	Reduces bias introduced by each decision tree through sequential construction of weak learners that adjust for misclassification from previous learners.
**Neural Network (Multilayer perceptron)**	• Predictors are represented as nodes.<br>• Signals from nodes are passed into a series of hidden node layers, each layer further abstracting the data by inducing nonlinearity and higher order interactions through the application of activation functions.<br>• Information from the hidden layers are transformed into an output layer that represents the class probabilities.
**Linear Discriminant Analysis**	• A dimensionality reduction approach which projects data to a lower dimensional space which minimizes within class variance and maximizes between class variance.<br>• Generative model which describes how original data is generated from lower dimensional information from each class.<br>• Lower dimensional information described with class-specific Gaussian distributions with class-specific priors. Shrinkage simplifies (i.e., makes more diagonal) covariance matrix estimated for large number of features to reduce overfitting.
**Quadratic Discriminant Analysis**	• Similar to Linear Discriminant Analysis.<br>• Co-variance estimation is not shared across classes.
**Support Vector Machine**	• Projects data into a higher dimensional space, constructed from application of kernel / similarity function on training data, that is more linearly separable (by hyper-plane estimated by maximizing distance to support vectors).<br>• Kernel functions calculate similarity between test set data and training instances to form test set predictions.
**Bayesian Logistic Regression**	• Similar to logistic regression.<br>• Samples posterior distribution of parameters by weighting likelihood function by prior distribution which reduces potential for bias. Similar to introducing L1/L2 penalty for traditional Logistic Regression.<br>• Many approaches exist for estimating the posterior distribution; we utilized stochastic variational inference (SVI) to approximate the posterior with a known family of distributions.
**Bayesian Additive Regression Trees (BART)**	• Successively samples decision trees, which, similar to XGBoost, adjust for errors introduced through previous trees, here through Bayesian backfitting. Estimated using a Particle Gibbs sampler which rejects trees based on Metropolis Hastings algorithm.<br>• Unlike XGBoost, priors are set over the tree structure/depth, selection of predictors, conditional means, and residual variance to penalize the model's complexity and limit bias. Samples from a posterior distribution of trees for any given tree iteration from the successive tree morphing.
**K-Nearest Neighbors**	Classifies observation through vote from class assignment of neighboring observations, as calculated through pairwise distance between observations and construction of a neighbor graph.

Details on Approaches to Limit Overfitting and Hyperparameter Scans

Some of the approaches listed in the previous section, if improperly tuned, can potentially be fit to memorize the exact relationship between each training observation and its corresponding outcome. This is referred to as overfitting, which can occur because certain machine learning models contain large enough information registries that are capable of storing the information imbued in each training instance and its corresponding outcome. Such a model would not generalize to unseen data and exhibit poor predictive performance. There are several approaches to combat overfitting, some

of which we employed in this work. For instance, introducing noise into the data (e.g., jitter the data by sampling from a gaussian distribution) can regularize the model, though we did not utilize this approach.

Two approaches we utilized to combat bias and limit overfitting were incorporation of Bayesian/penalization methods (see above) and, to identify the optimal penalization, implementation of 5-fold cross validation for hyperparameter scanning. Cross validation subdivides the training/validation dataset into training and validation folds (here selected at 80%/20% subpartitions). For each fold, models are fit to the training data and evaluated (i.e., return an AUC) on "held-out" validation data to estimate how the model may perform on a held-out test dataset. This process is repeated for the four other training/validation folds to form an unbiased estimate of the capacity of the model to generalize to unseen data. Machine learning models commonly employ "hyperparameters" which specify their configuration (e.g., number of trees in forest, tree depth), that if left unmodified, may be overfit. Cross validated performance metrics averaged across the validation folds are used to compare model performance across different sets of hyperparameters to select a set which will likely perform the best on the held-out test data. The model is retrained on the entire training/validation dataset using the hyperparameter set which performed best and evaluated on the test set. For the held-out dataset and cross validation training and validation folds, we maintained the proportion of patients with HAPI (stratified 5-fold cross validation). We also ensured folds contained the same set of patients for training and evaluating each approach.

Below, we have created a table which enumerates a grid of hyperparameters, all combinations of which were compared during the hyperparameter scans. We bolded hyperparameters that were ultimately selected for each modeling approach. Note that unlisted hyperparameters were set to the default by the relevant software package.

Supplementary Table 7: Detailed description of hyperparameter grid for hyperparameter scan; Selected hyperparameters are bolded

Approach	Brief Description
**Logistic Regression**	• Penalty: **None**, L1, L2<br>• Penalization Strength: **0**, 1e-4, 1e-3, 1e-2, 1e-1, 1, 10, 100
**Naive Bayes**	• **None required**
**Decision Trees**	• Maximum tree depth: 3, 5, 8, 10, 15, **20**, None<br>• Minimum proportion of samples to split on: 0.2, 0.4, **0.6**<br>• Minimum proportion of samples for leaf: 0.2, **0.4**, 0.6<br>• Maximum number of selected features for tree: Log2(p), Sqrt(p), **24**
**Random Forest**	• Maximum tree depth: 3, 4, 5, 8, 10, 15, **20**, None<br>• Number of estimators: 10, 20, 40, 60, **80**, 100<br>• Maximum number of selected features for tree: Log2(p), **Sqrt(p)**
**XGBoost**	• Maximum tree depth: **2**, 4, 6, 8, 10, None<br>• Number of estimators: 10, 20, 40, 60, 80, **100**<br>• Minimum number of samples to split on: 1, 3, **5**, 7<br>• Learning rate: 1e-4, 1e-3, 1e-2, **1e-1**

**Neural Network (Multilayer perceptron)**	• Hidden layer architecture: **[100]**, [30,30], [30,200,30]<br>• Weight decay (L2 penalty): **0.0001**, 0.001, 0.01<br>• Initial learning rate: 1e-1,1e-2,**1e-3**
**Linear Discriminant Analysis**	• Shrinkage: **None**, Automatic, 0.1, 0.5, 0.8
**Quadratic Discriminant Analysis**	• Regularization parameter: **0**, 0.2, 0.5, 0.8, 1
**Support Vector Machine**	• Penalty: L1, **L2**, None<br>• Penalization Strength: 0.01,0.1,**1**,10,100
**Bayesian Logistic Regression**	• Number of Posterior Samples: 1000, 5000, 10000, **20000**<br>• Learning Rate: **1**,1e-1,1e-2<br>• Batch Size: 500,1000,**1500**,2500<br>• Intercept prior standard deviation: 1, 10, **100**, 1000<br>• Parameter prior standard deviation: 1, **10**, 100,1000
**Bayesian Additive Regression Trees (BART)**	• Number of trees: 10, 20, 30, **50**, 70<br>• Tree depth prior: **0.3**, 0.5, 0.7<br>• Number of posterior samples for each tree: 10, **15**, 50, 75
**K-Nearest Neighbors**	• Number of neighbors: 5,10,**25**,35,65,75,100

Circumventing Class Imbalance Issues

In accordance with a recent pressure injury modeling study that found under-sampling negative samples to be an effective modeling technique [1], we employed techniques to upweight the importance of the positive samples [2]. For logistic regression, this meant assigning a higher weight to the positive class, while for random forest techniques, this meant under-sampling the occurrence of negative controls during training time. Experiments with other class balancing techniques such as oversampling and SMOTE (Synthetic Minority Over-Sampling Technique, which over-samples the minority class) [3] appeared to not be as effective as reweighting the model objective and under-sampling during training. Preliminary testing of this technique demonstrated that adding the class balancing weight marginally improved the AUC of the resultant model for the test data set (selected with the same class imbalance as the training set). Logistic regression techniques are well-equipped to handle rare events and thus do not usually require class balancing. Re-weighting the Bernoulli likelihood of the logistic regression model based on the rarity of a particular class should lead to equivalent results as an “unbalanced” model with the artifact of skewing the derived odds ratio estimates. However, other machine learning models may not explicitly account for rare events and thus require class balancing. To this end, we implemented these class balancing techniques for all models to offer a fair comparison. In addition, proportions of HAPI to control cases were maintained across all cross-validation folds.

Supplementary Findings from All Modeling Approaches

Supplementary Table 8: Reported AUCs for each cross-validation fold for each modeling approach and average AUC across the folds; 95% confidence intervals obtained using 1000 non-parametric bootstrapped resamplings with replacement; using same sample of patients for each bootstrap iteration to calculate average AUC

Model	Val-Fold 1 AUC	Val-Fold 2 AUC	Val-Fold 3 AUC	Val-Fold 4 AUC	Val-Fold 5 AUC	Average Val AUC	2.5% CI	97.5% CI
**Support Vector Machine**	0.921	0.928	0.892	0.857	0.938	0.908	0.887	0.925
**Neural Network**	0.916	0.933	0.894	0.856	0.939	0.908	0.890	0.925
**K Nearest Neighbors**	0.772	0.787	0.773	0.702	0.852	0.778	0.744	0.811
**Linear Discriminant Analysis**	0.895	0.920	0.886	0.846	0.929	0.896	0.874	0.913
**Quadratic Discriminant Analysis**	0.865	0.875	0.863	0.876	0.912	0.879	0.852	0.900
**Logistic Regression**	0.908	0.917	0.893	0.858	0.936	0.903	0.882	0.920
**Naive Bayes**	0.861	0.917	0.880	0.850	0.921	0.886	0.865	0.906
**Decision Tree**	0.737	0.783	0.768	0.712	0.778	0.757	0.737	0.774
**Random Forest**	0.896	0.936	0.896	0.842	0.926	0.900	0.879	0.919
**XGBoost**	0.899	0.927	0.886	0.850	0.933	0.899	0.880	0.918
**Bayesian Logistic Regression**	0.907	0.929	0.896	0.869	0.936	0.908	0.890	0.925
**BART**	0.912	0.931	0.914	0.879	0.945	0.917	0.899	0.932

Supplementary Table 9: Comparison of AUCs between modeling approaches averaged across five cross validation folds; 95% confidence intervals obtained using 1000 non-parametric bootstrapped resamplings with replacement; using same sample of patients for each bootstrap iteration to calculate difference in average AUC

Model 1	Model 2	ΔAUC Estimate	2.5% CI	97.5% CI	Reject H0
**Naive Bayes**	**Neural Network**	-0.022	-0.036	-0.007	TRUE
**BART**	**Neural Network**	0.008	0.001	0.018	TRUE
**Linear Discriminant Analysis**	**Naive Bayes**	0.009	-0.007	0.025	FALSE
**Bayesian Logistic Regression**	**XGBoost**	0.009	-0.001	0.019	FALSE
**Decision Tree**	**K Nearest Neighbors**	-0.022	-0.053	0.011	FALSE
**Support Vector Machine**	**XGBoost**	0.008	-0.001	0.018	FALSE
**Logistic Regression**	**Neural Network**	-0.005	-0.015	0.003	FALSE
**K Nearest Neighbors**	**Linear Discriminant Analysis**	-0.118	-0.152	-0.087	TRUE
**Linear Discriminant Analysis**	**Support Vector Machine**	-0.012	-0.020	-0.004	TRUE
**Decision Tree**	**Linear Discriminant Analysis**	-0.139	-0.160	-0.121	TRUE
**Decision Tree**	**Logistic Regression**	-0.147	-0.164	-0.130	TRUE
**Linear Discriminant Analysis**	**Logistic Regression**	-0.007	-0.017	0.003	FALSE
**Logistic Regression**	**Naive Bayes**	0.016	0.002	0.032	TRUE
**Naive Bayes**	**Support Vector Machine**	-0.021	-0.037	-0.005	TRUE
**K Nearest Neighbors**	**Neural Network**	-0.130	-0.160	-0.102	TRUE
**Bayesian Logistic Regression**	**Neural Network**	-0.000	-0.007	0.008	FALSE

**Decision Tree**	**XGBoost**	-0.143	-0.162	-0.124	TRUE
**Bayesian Logistic Regression**	**Linear Discriminant Analysis**	0.012	0.001	0.024	TRUE
**Naive Bayes**	**XGBoost**	-0.013	-0.029	0.002	FALSE
**BART**	**Linear Discriminant Analysis**	0.021	0.010	0.033	TRUE
**Quadratic Discriminant Analysis**	**Random Forest**	-0.021	-0.043	-0.001	TRUE
**BART**	**XGBoost**	0.017	0.008	0.028	TRUE
**Linear Discriminant Analysis**	**Quadratic Discriminant Analysis**	0.017	-0.002	0.039	FALSE
**K Nearest Neighbors**	**Random Forest**	-0.121	-0.153	-0.093	TRUE
**Linear Discriminant Analysis**	**Random Forest**	-0.004	-0.017	0.008	FALSE
**BART**	**Logistic Regression**	0.014	0.005	0.024	TRUE
**Bayesian Logistic Regression**	**Logistic Regression**	0.005	-0.004	0.015	FALSE
**Decision Tree**	**Quadratic Discriminant Analysis**	-0.122	-0.146	-0.097	TRUE
**K Nearest Neighbors**	**XGBoost**	-0.121	-0.153	-0.093	TRUE
**K Nearest Neighbors**	**Support Vector Machine**	-0.130	-0.163	-0.098	TRUE
**Decision Tree**	**Random Forest**	-0.143	-0.158	-0.129	TRUE
**Bayesian Logistic Regression**	**Quadratic Discriminant Analysis**	0.029	0.014	0.049	TRUE
**Linear Discriminant Analysis**	**XGBoost**	-0.004	-0.017	0.009	FALSE
**Naive Bayes**	**Quadratic Discriminant Analysis**	0.007	-0.007	0.024	FALSE
**Bayesian Logistic Regression**	**Random Forest**	0.008	-0.003	0.020	FALSE
**Naive Bayes**	**Random Forest**	-0.013	-0.028	0.000	FALSE
**Quadratic Discriminant Analysis**	**Support Vector Machine**	-0.029	-0.048	-0.012	TRUE
**Neural Network**	**Support Vector Machine**	0.000	-0.007	0.008	FALSE
**BART**	**K Nearest Neighbors**	0.138	0.111	0.170	TRUE
**Decision Tree**	**Naive Bayes**	-0.131	-0.147	-0.112	TRUE
**BART**	**Decision Tree**	0.161	0.146	0.176	TRUE
**BART**	**Quadratic Discriminant Analysis**	0.038	0.020	0.059	TRUE
**Bayesian Logistic Regression**	**Support Vector Machine**	0.000	-0.008	0.009	FALSE
**Bayesian Logistic Regression**	**K Nearest Neighbors**	0.130	0.102	0.161	TRUE
**Neural Network**	**Quadratic Discriminant Analysis**	0.029	0.013	0.048	TRUE
**BART**	**Random Forest**	0.017	0.008	0.027	TRUE
**Neural Network**	**Random Forest**	0.009	-0.002	0.019	FALSE
**Logistic Regression**	**Quadratic Discriminant Analysis**	0.024	0.008	0.043	TRUE
**BART**	**Naive Bayes**	0.031	0.018	0.045	TRUE
**Random Forest**	**XGBoost**	0.000	-0.011	0.012	FALSE
**K Nearest Neighbors**	**Naive Bayes**	-0.108	-0.138	-0.078	TRUE
**BART**	**Support Vector Machine**	0.009	0.000	0.019	TRUE
**Neural Network**	**XGBoost**	0.009	0.000	0.018	TRUE
**Quadratic Discriminant Analysis**	**XGBoost**	-0.021	-0.040	-0.004	TRUE
**Bayesian Logistic Regression**	**BART**	-0.009	-0.017	-0.001	TRUE
**Decision Tree**	**Neural Network**	-0.152	-0.169	-0.134	TRUE
**Random Forest**	**Support Vector Machine**	-0.008	-0.018	0.004	FALSE
**Logistic Regression**	**Random Forest**	0.003	-0.007	0.013	FALSE
**Logistic Regression**	**Support Vector Machine**	-0.005	-0.012	0.002	FALSE
**Logistic Regression**	**XGBoost**	0.003	-0.006	0.013	FALSE
**Bayesian Logistic Regression**	**Decision Tree**	0.152	0.134	0.169	TRUE
**Linear Discriminant Analysis**	**Neural Network**	-0.012	-0.024	-0.001	TRUE
**K Nearest Neighbors**	**Quadratic Discriminant Analysis**	-0.100	-0.132	-0.069	TRUE
**Decision Tree**	**Support Vector Machine**	-0.151	-0.170	-0.133	TRUE
**K Nearest Neighbors**	**Logistic Regression**	-0.125	-0.157	-0.096	TRUE
**Bayesian Logistic Regression**	**Naive Bayes**	0.022	0.010	0.033	TRUE

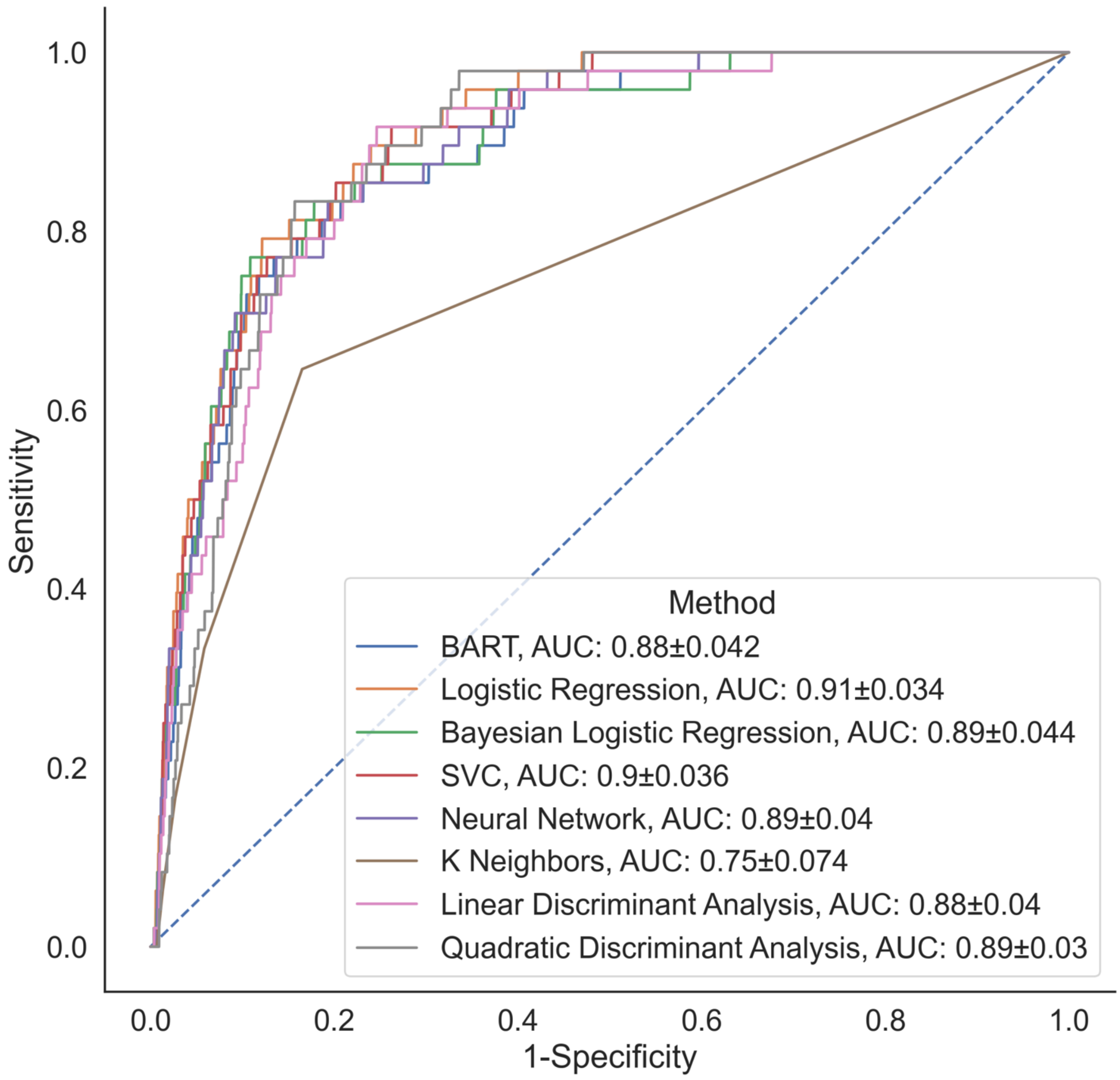

Supplementary Figure 2: Held-out test set AUC results for additional modeling approaches

Supplementary Table 10: Reported AUCs for each modeling approach on the held out test set; 95% confidence intervals obtained using 1000 non-parametric bootstrapped resamplings with replacement

Model	AUC Estimate	2.5% AUC	97.5% AUC
**Support Vector Machine**	0.899	0.860	0.930
**Neural Network**	0.890	0.849	0.925
**K Nearest Neighbors**	0.751	0.676	0.816

**Linear Discriminant Analysis**	0.884	0.839	0.917
**Quadratic Discriminant Analysis**	0.891	0.857	0.917
**Logistic Regression**	0.907	0.869	0.935
**Naive Bayes**	0.866	0.829	0.898
**Decision Tree**	0.759	0.716	0.792
**Random Forest**	0.897	0.858	0.926
**XGBoost**	0.888	0.846	0.924
**Bayesian Logistic Regression**	0.890	0.843	0.926
**BART**	0.885	0.841	0.920

Supplementary Table 11: Comparison of AUCs between modeling approaches on the held-out test set; 95% confidence intervals obtained using 1000 non-parametric bootstrapped resamplings with replacement; using same sample of patients for each bootstrap iteration to calculate difference in AUC

**Model 1**	**Model 2**	**ΔAUC Estimate**	**2.5% CI**	**97.5% CI**	**Reject H0**
**Naive Bayes**	**Neural Network**	-0.024	-0.049	0.004	FALSE
**Bayesian Logistic Regression**	**XGBoost**	0.002	-0.033	0.036	FALSE
**Linear Discriminant Analysis**	**Naive Bayes**	0.017	-0.007	0.044	FALSE
**Decision Tree**	**K Nearest Neighbors**	0.008	-0.052	0.078	FALSE
**Support Vector Machine**	**XGBoost**	0.011	-0.010	0.037	FALSE
**Logistic Regression**	**Neural Network**	0.016	0.005	0.029	TRUE
**K Nearest Neighbors**	**Linear Discriminant Analysis**	-0.134	-0.195	-0.079	TRUE
**Linear Discriminant Analysis**	**Support Vector Machine**	-0.016	-0.031	0.002	FALSE
**BART**	**K Nearest Neighbors**	0.134	0.072	0.199	TRUE
**Decision Tree**	**Linear Discriminant Analysis**	-0.125	-0.154	-0.098	TRUE
**Decision Tree**	**Logistic Regression**	-0.148	-0.179	-0.115	TRUE
**BART**	**Bayesian Logistic Regression**	-0.006	-0.024	0.011	FALSE
**Linear Discriminant Analysis**	**Logistic Regression**	-0.023	-0.044	0.001	FALSE
**Logistic Regression**	**Naive Bayes**	0.041	0.016	0.065	TRUE
**Naive Bayes**	**Support Vector Machine**	-0.033	-0.059	-0.009	TRUE
**K Nearest Neighbors**	**Neural Network**	-0.140	-0.202	-0.081	TRUE
**Bayesian Logistic Regression**	**Neural Network**	-0.000	-0.013	0.012	FALSE
**Decision Tree**	**XGBoost**	-0.129	-0.151	-0.104	TRUE
**BART**	**Decision Tree**	0.126	0.090	0.157	TRUE
**Bayesian Logistic Regression**	**Linear Discriminant Analysis**	0.008	-0.030	0.037	FALSE
**Naive Bayes**	**XGBoost**	-0.021	-0.048	0.006	FALSE

**Quadratic Discriminant Analysis**	**Random Forest**	-0.005	-0.026	0.017	FALSE
**BART**	**Support Vector Machine**	-0.015	-0.035	0.002	FALSE
**Linear Discriminant Analysis**	**Quadratic Discriminant Analysis**	-0.007	-0.035	0.018	FALSE
**BART**	**Naive Bayes**	0.018	-0.008	0.044	FALSE
**K Nearest Neighbors**	**Random Forest**	-0.146	-0.208	-0.091	TRUE
**Linear Discriminant Analysis**	**Random Forest**	-0.012	-0.032	0.008	FALSE
**Bayesian Logistic Regression**	**Logistic Regression**	-0.016	-0.035	-0.001	TRUE
**Decision Tree**	**Quadratic Discriminant Analysis**	-0.133	-0.165	-0.099	TRUE
**K Nearest Neighbors**	**XGBoost**	-0.138	-0.202	-0.080	TRUE
**K Nearest Neighbors**	**Support Vector Machine**	-0.150	-0.211	-0.094	TRUE
**Decision Tree**	**Random Forest**	-0.137	-0.157	-0.117	TRUE
**BART**	**Random Forest**	-0.011	-0.033	0.007	FALSE
**Bayesian Logistic Regression**	**Quadratic Discriminant Analysis**	-0.000	-0.032	0.025	FALSE
**BART**	**Linear Discriminant Analysis**	0.001	-0.030	0.027	FALSE
**Linear Discriminant Analysis**	**XGBoost**	-0.004	-0.027	0.020	FALSE
**Naive Bayes**	**Quadratic Discriminant Analysis**	-0.025	-0.049	-0.003	TRUE
**Bayesian Logistic Regression**	**Random Forest**	-0.005	-0.037	0.021	FALSE
**Naive Bayes**	**Random Forest**	-0.029	-0.049	-0.012	TRUE
**Quadratic Discriminant Analysis**	**Support Vector Machine**	-0.008	-0.030	0.014	FALSE
**Neural Network**	**Support Vector Machine**	-0.009	-0.025	0.003	FALSE
**BART**	**Logistic Regression**	-0.021	-0.043	-0.003	TRUE
**Decision Tree**	**Naive Bayes**	-0.108	-0.135	-0.076	TRUE
**BART**	**Quadratic Discriminant Analysis**	-0.007	-0.033	0.017	FALSE
**Bayesian Logistic Regression**	**Support Vector Machine**	-0.008	-0.030	0.008	FALSE
**Bayesian Logistic Regression**	**K Nearest Neighbors**	0.140	0.076	0.207	TRUE
**Neural Network**	**Quadratic Discriminant Analysis**	-0.001	-0.028	0.022	FALSE
**Neural Network**	**Random Forest**	-0.005	-0.030	0.013	FALSE
**Logistic Regression**	**Quadratic Discriminant Analysis**	0.015	-0.006	0.037	FALSE
**Random Forest**	**XGBoost**	0.008	-0.008	0.024	FALSE
**BART**	**XGBoost**	-0.003	-0.026	0.016	FALSE
**K Nearest Neighbors**	**Naive Bayes**	-0.116	-0.178	-0.064	TRUE
**Neural Network**	**XGBoost**	0.003	-0.025	0.027	FALSE
**Quadratic Discriminant Analysis**	**XGBoost**	0.003	-0.026	0.034	FALSE
**BART**	**Neural Network**	-0.006	-0.021	0.009	FALSE
**Decision Tree**	**Neural Network**	-0.132	-0.164	-0.094	TRUE
**Random Forest**	**Support Vector Machine**	-0.003	-0.020	0.012	FALSE
**Logistic Regression**	**Random Forest**	0.011	-0.009	0.031	FALSE

**Logistic Regression**	**Support Vector Machine**	0.007	-0.002	0.016	FALSE
**Logistic Regression**	**XGBoost**	0.018	-0.007	0.047	FALSE
**Bayesian Logistic Regression**	**Decision Tree**	0.132	0.086	0.172	TRUE
**Linear Discriminant Analysis**	**Neural Network**	-0.007	-0.030	0.023	FALSE
**K Nearest Neighbors**	**Quadratic Discriminant Analysis**	-0.142	-0.207	-0.079	TRUE
**Decision Tree**	**Support Vector Machine**	-0.140	-0.170	-0.111	TRUE
**K Nearest Neighbors**	**Logistic Regression**	-0.157	-0.219	-0.100	TRUE
**Bayesian Logistic Regression**	**Naive Bayes**	0.025	-0.008	0.055	FALSE

Comparison of SHAP versus LIME Interpretations on a Few Select Cases

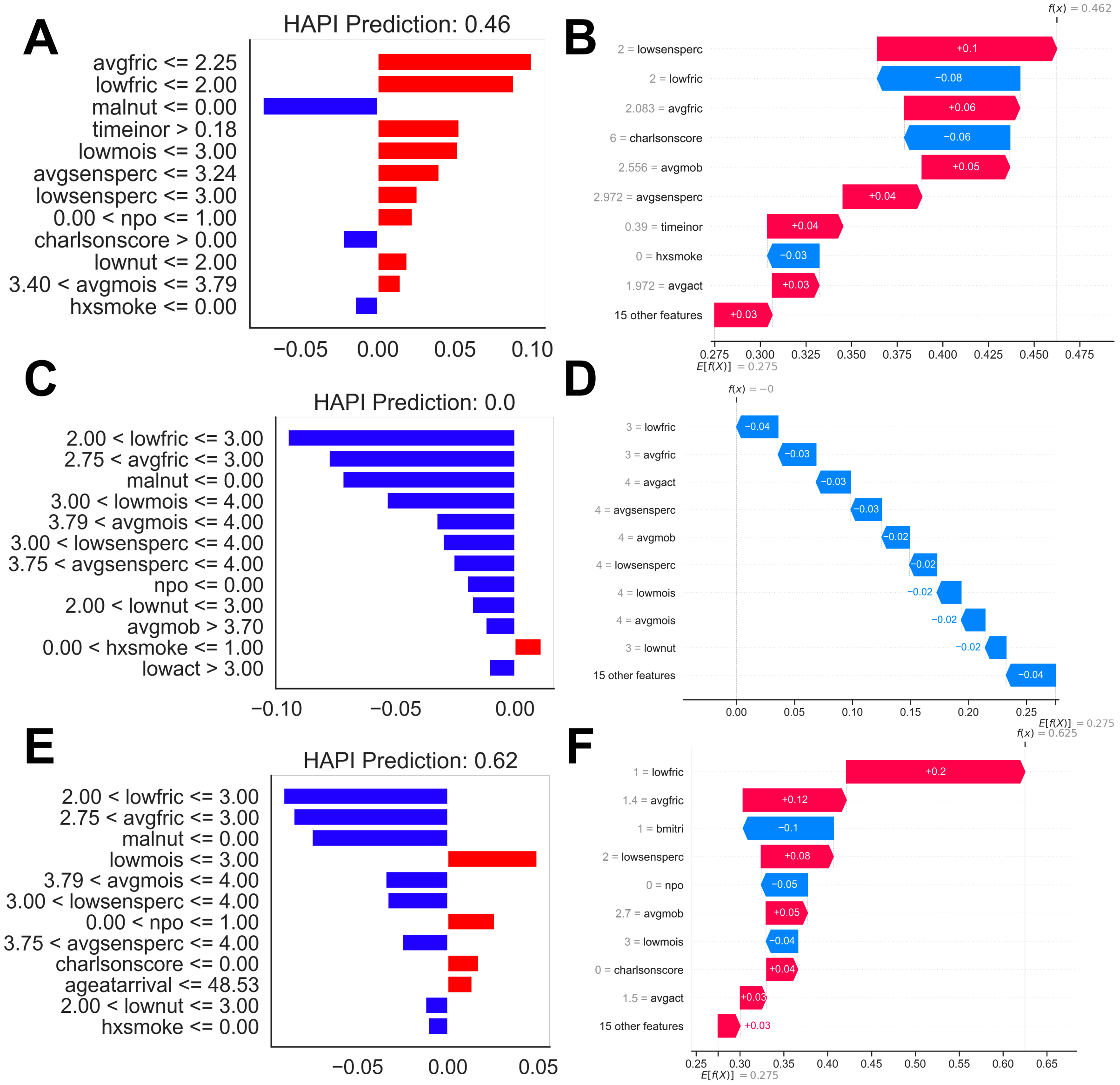

Supplementary Figure 3: SHAP versus LIME on a few randomly selected cases: Model importances (red for HAPI-associated; blue for control-associated) were reported for the random forest estimator for (A,C,E) LIME, and (B,D,F) SHAP via waterfall plots; general concordance is noted for patients 1 (A,B) and 2 (C,D), though findings differ significantly for patient 3 (E,F); note that predictor values are communicated after standardizing the predictors

SHAP Comparisons between Logistic Regression, Random Forest, XGBoost

Supplementary Table 12: Spearman's rank correlation coefficients and rank biased overlap (RBO) scores between important features of Random Forest (RF), XGBoost (XG) and Logistic Regression (LR); 95% confidence intervals calculated using 1000 nonparametric bootstrap resampling of Shapley values with replacement prior to calculation of global feature importances; RBO's decay term set to 0.9 to weight successive important terms 90% from the previous term's importance for calculation of feature importance

	Statistic	Estimate	2.5% CI	97.5% CI
**Spearman Correlation Coefficient**	$\rho_{XG,LR}$	0.706	0.690	0.713
	$\rho_{RF,LR}$	0.732	0.724	0.747
	$\rho_{RF,XG}$	0.760	0.748	0.772
	$\rho_{RF,XG} - \rho_{RF,LR}$	0.055	0.040	0.070
	$\rho_{RF,XG} - \rho_{XG,LR}$	0.024	0.012	0.037
	$\rho_{RF,LR} - \rho_{XG,LR}$	0.028	0.016	0.051
**Rank Biased Overlap (p=0.9)**	$\rho_{XG,LR}$	0.683	0.679	0.683
	$\rho_{RF,LR}$	0.638	0.637	0.645
	$\rho_{RF,XG}$	0.670	0.666	0.672
	$\rho_{RF,XG} - \rho_{RF,LR}$	-0.013	-0.017	-0.009
	$\rho_{RF,XG} - \rho_{XG,LR}$	0.030	0.026	0.033
	$\rho_{RF,LR} - \rho_{XG,LR}$	-0.045	-0.049	-0.034

Supplementary Table 13: Ranking of overall important features for each modeling approach. Lower ranks constitute important features.

	Logistic Regression	Random Forest	XG-Boost
**lowfric**	1	1	3
**avgmob**	2	3	2
**avgfric**	3	8	1
**lownut**	4	19	10
**npo**	5	4	6
**avgact**	6	7	12
**avgmois**	7	16	19
**lowsensperc**	8	2	16
**lowmois**	9	9	7
**avgnut**	10	11	5

**lowmob**	11	10	22
**charlsonscore**	12	22	8
**malnut**	13	6	13
**hxsmoke**	14	5	4
**lowact**	15	15	9
**timeinor**	16	13	20
**white**	17	14	11
**admit_icu**	18	20	21
**avgsensperc**	19	12	15
**bmitri**	20	23	17
**male**	21	24	14
**ageatarrival**	22	21	23
**ambulate**	23	18	18
**bedrest**	24	17	24

Supplementary Table 14: Global Feature Importance; Random Forest Values are significantly lower because SHAP values are to be interpreted as an increase or decrease in HAPI probability, while SHAP values for Logistic Regression and XG-Boost are to be interpreted as a Log-Odds

	SHAP Logistic Regression	Random Forest	XG-Boost	Logistic Regression Standardized Coefficients
lowfric	1.07	0.06	0.44	1.22
avgmob	0.43	0.02	0.51	0.45
avgfric	0.39	0.04	0.58	0.41
lownut	0.39	0.03	0.08	0.56
npo	0.38	0.01	0.13	0.23
avgact	0.36	0.01	0.20	0.36
avgmois	0.28	0.02	0.14	0.17
lowsensperc	0.27	0.03	0.09	0.29
lowmois	0.26	0.01	0.03	0.19
avgnut	0.26	0.01	0.37	0.32
lowmob	0.20	0.01	0.01	0.03
charlsonscore	0.19	0.00	0.19	0.36
malnut	0.16	0.01	0.09	0.20
hxsmoke	0.16	0.00	0.00	0.01
lowact	0.14	0.01	0.00	0.25
timeinor	0.12	0.02	0.17	0.16
white	0.05	0.00	0.00	0.04
admit_icu	0.05	0.00	0.00	0.06
avgsensperc	0.03	0.03	0.19	0.14
bmitri	0.03	0.00	0.02	0.04
male	0.02	0.00	0.00	0.07
ageatarrival	0.02	0.01	0.11	0.06
ambulate	0.01	0.00	0.00	0.07
bedrest	0.01	0.00	0.00	0.02

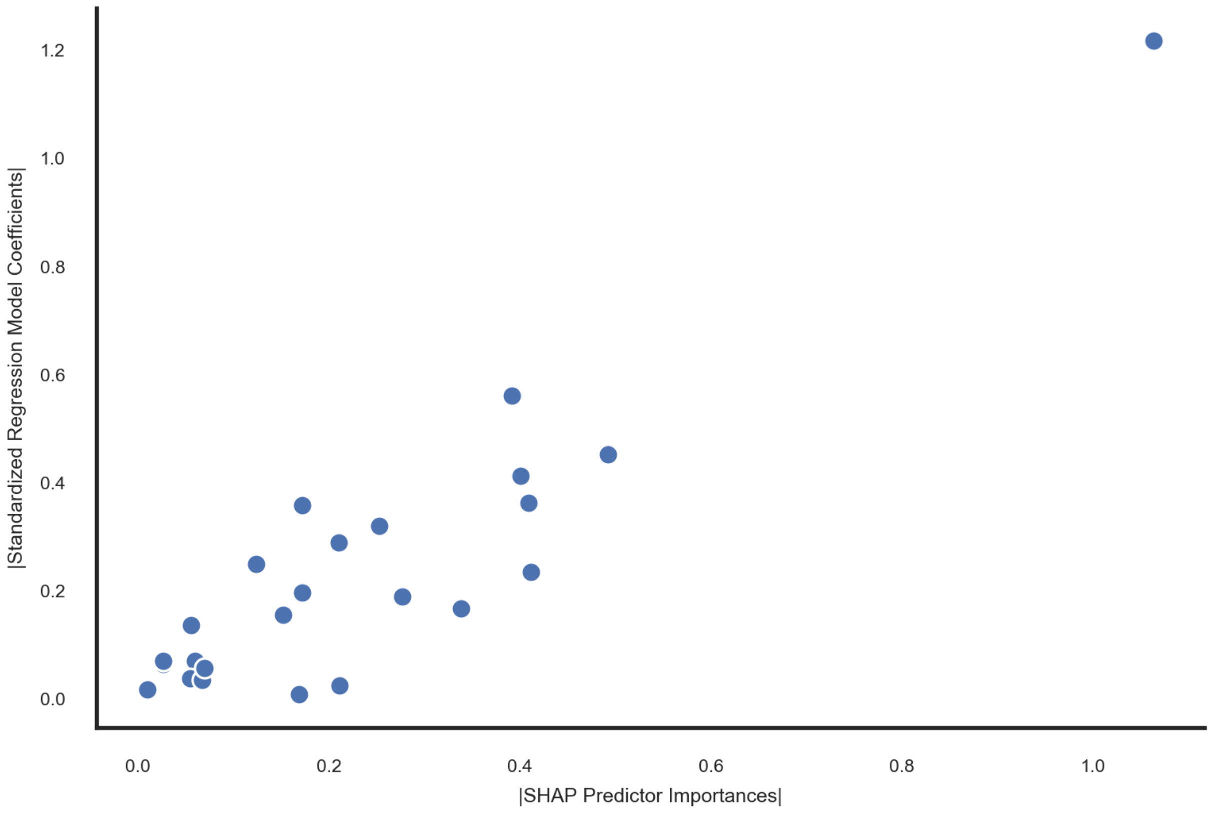

Supplementary Figure 4: Agreement between SHAP and standardized regression coe fficients (r=0.914, p=4.5e-10)

Supplementary Table 15: Standardized Regression Coefficients of Logistic Regression Model

	coef	std err	z	P>|z|	[0.025	0.975]
**const**	-1.7	1.0	-1.7	0.1	-3.7	0.3
**ageatarrival**	0.0	0.0	0.7	0.5	-0.0	0.0
**male**	0.1	0.2	0.9	0.4	-0.2	0.4
**white**	-0.3	0.5	-0.5	0.6	-1.2	0.7
**hxsmoke**	0.0	0.2	0.1	0.9	-0.3	0.3
**bmitri**	-0.1	0.2	-0.5	0.6	-0.4	0.2
**admit_icu**	-0.2	0.2	-0.9	0.4	-0.5	0.2
**ambulate**	0.2	0.2	0.9	0.4	-0.2	0.6
**bedrest**	-0.0	0.2	-0.2	0.8	-0.4	0.3
**npo**	0.5	0.3	1.7	0.1	-0.1	1.1
**timeinor**	1.4	0.4	3.5	0.0	0.6	2.2
**malnut**	0.7	0.2	4.3	0.0	0.4	1.1
**charlsonscore**	-0.2	0.1	-3.4	0.0	-0.4	-0.1
**avgmois**	0.4	0.3	1.5	0.1	-0.1	1.0
**avgsensperc**	0.3	0.2	1.1	0.3	-0.2	0.7

**avgact**	0.5	0.2	2.0	0.0	0.0	0.9
**avgmob**	-0.8	0.3	-2.4	0.0	-1.4	-0.1
**avgnut**	0.6	0.2	2.8	0.0	0.2	1.1
**avgfric**	-0.9	0.3	-3.2	0.0	-1.5	-0.4
**lowmois**	-0.3	0.1	-2.0	0.0	-0.5	-0.0
**lowsensperc**	-0.3	0.2	-2.0	0.0	-0.6	-0.0
**lowact**	0.2	0.2	1.3	0.2	-0.1	0.6
**lowmob**	0.0	0.2	0.2	0.9	-0.3	0.4
**lownut**	-0.7	0.2	-3.8	0.0	-1.1	-0.4
**lowfric**	-1.8	0.2	-7.8	0.0	-2.3	-1.4

Supplementary Table 16: Regression Coefficients of Logistic Regression Model

	**coef**	**std err**	**z**	**P>|z|**	**[0.025**	**0.975]**
**const**	-7.4	0.2	-35.7	0.0	-7.8	-7.0
**ageatarrival**	0.1	0.1	0.7	0.5	-0.1	0.2
**male**	0.1	0.1	0.9	0.4	-0.1	0.2
**white**	-0.0	0.1	-0.5	0.6	-0.2	0.1
**hxsmoke**	0.0	0.1	0.1	0.9	-0.1	0.2
**bmitri**	-0.0	0.1	-0.5	0.6	-0.2	0.1
**admit_icu**	-0.1	0.1	-0.9	0.4	-0.2	0.1
**ambulate**	0.1	0.1	0.9	0.4	-0.1	0.2
**bedrest**	-0.0	0.1	-0.2	0.8	-0.2	0.1
**npo**	0.2	0.1	1.7	0.1	-0.0	0.5
**timeinor**	0.2	0.0	3.5	0.0	0.1	0.2
**malnut**	0.2	0.0	4.3	0.0	0.1	0.3
**charlsonscore**	-0.4	0.1	-3.4	0.0	-0.6	-0.2
**avgmois**	0.2	0.1	1.5	0.1	-0.1	0.4
**avgsensperc**	0.1	0.1	1.1	0.3	-0.1	0.4
**avgact**	0.4	0.2	2.0	0.0	0.0	0.7
**avgmob**	-0.5	0.2	-2.4	0.0	-0.8	-0.1
**avgnut**	0.3	0.1	2.8	0.0	0.1	0.5
**avgfric**	-0.4	0.1	-3.2	0.0	-0.7	-0.2
**lowmois**	-0.2	0.1	-2.0	0.0	-0.4	-0.0
**lowsensperc**	-0.3	0.1	-2.0	0.0	-0.6	-0.0
**lowact**	0.2	0.2	1.3	0.2	-0.1	0.6
**lowmob**	0.0	0.2	0.2	0.9	-0.3	0.3
**lownut**	-0.6	0.1	-3.8	0.0	-0.8	-0.3
**lowfric**	-1.2	0.2	-7.8	0.0	-1.5	-0.9

Extended Discussion on SHAP Limitations
Here, we provide an extended discussion on a few additional limitations of the SHAP approach:

Inconsistent Causal Interpretations: SHAP models have been shown to have inconsistent causal interpretations. When interpreting the model, a predictor can indirectly influence the outcome, while other predictor variables may drive the relationship (i.e., the data generating mechanism). This can partly be attributed to the fact that relations between different predictors may not be well understood as suggested by our earlier discussion. This understanding can impact how findings are interpreted (i.e., should the clinician intervene on an identified predictor that has little causal influence?). While SHAP applied to Logistic Regression produced findings similar to the logistic regression coefficients, both should be interpreted within the causal constraints allowed by the assumed data generating mechanism. Working with the domain expert (i.e., clinical stakeholder) is crucial to inform a working causal model.

Interpretation is with respect to a selected baseline: Typically, this is selected *ad hoc* by the conditional mean of the prediction function. The additive model attempts to explain the difference between the baseline and the prediction which could be problematic in imbalanced settings.

Interpretation is dependent on the interpretability approach: The SHAP library allows one to estimate SHAP values based on "interventional" versus "observational" distributions; the former of which ignores correlations between features and is thus provides limited causal input but is commonly employed due to the speed of computation, while the latter does account for such correlations to provide a more causal interpretation, better capturing the data generating mechanism.

SHAP does not reflect the user's freedom of choice: SHAP in its current formulation assumes that an individual would select the explanation as a plausible alternative amongst a set of theoretically generated alternatives and counterfactual explanations. Individuals from different backgrounds and perspectives may be more predisposed (whether based on upbringing, underrepresentation, background, training, etc.) to consider a different list of alternative explanations that connects with their ability to make rapid comparisons.

Incorporation of fairness-based explanation approaches (which may extrapolate out of distribution and communicate uncertainty in cases where underrepresented groups are largely omitted from a predictive approach), in tandem with stakeholder engagement, can help craft decision aids that can make best use of model explanation techniques by considering a pluralism of different stakeholder perspectives. Note that we did not perform user testing with SHAP to see if use of the approach would reduce pressure injuries. We plan to follow up this study with real-world testing that is sensitive to potential ethical dilemmas introduced in the main text.

References

1. Nakamura Y, Ghaibeh AA, Setoguchi Y, Mitani K, Abe Y, Hashimoto I, Moriguchi H (2015) On-Admission Pressure Ulcer Prediction Using the Nursing Needs Score. JMIR Med Inform. https://doi.org/10.2196/medinform.3850

2. Longadge R, Dongre S (2013) Class Imbalance Problem in Data Mining Review. Int. J. Comput. Sci. Netw. 2:

3. Fernandez A, Garcia S, Herrera F, Chawla NV (2018) SMOTE for Learning from Imbalanced Data: Progress and Challenges, Marking the 15-year Anniversary. Journal of Artificial Intelligence Research 61:863–905
